# Joint Task Offloading and Resource Allocation with Data Caching in UAV-Aided Mobile Edge Computing Networks for Latency-Sensitive Applications

**DOI:** 10.3390/s26154966

**Published:** 2026-08-05

**Authors:** Tanmay Baidya, Sangman Moh

**Affiliations:** Department of Computer Engineering, Chosun University, 309 Pilmun-daero, Dong-gu, Gwangju 61452, Republic of Korea

**Keywords:** caching, deep reinforcement learning, mobile edge computing, offloading, resource allocation, soft actor–critic, unmanned aerial vehicle

## Abstract

The rapid growth of computing-intensive and latency-sensitive applications, including augmented reality, virtual reality, and self-driving systems, has increased the demand for low-latency and energy-efficient processing solutions. Mobile edge computing (MEC) has evolved as a transformative paradigm by relocating computation to the network edge, closer to end users. Unmanned aerial vehicles (UAVs) further strengthen MEC by offering flexible deployment, mobility, and reliable line-of-sight communication, making them suitable for temporary high-demand scenarios. Moreover, such latency-sensitive applications often generate numerous repetitive tasks and, thus, storing the results of these tasks can reduce both communication overhead and computational workload. However, jointly addressing the caching of task-results alongside offloading and resource allocation decisions in UAV-aided MEC networks remains a non-trivial challenge. In this study, an integrated task offloading and resource allocation with data caching (JORC) framework is proposed to address these challenges. The offloading and resource allocation problems are formulated as a Markov decision process and solved using the soft actor–critic reinforcement learning algorithm. In addition, dynamic and adaptive caching manages limited storage and reduces redundant computations by using a hybrid strategy that integrates the least-frequently used and least-recently used policies to reduce computational redundancy. Simulation results confirm that the proposed JORC framework substantially reduces latency, energy consumption, and overall system cost, while increasing the successful task completion ratio compared to existing baseline approaches.

## 1. Introduction

The rapid advancement of latency-sensitive and computation-intensive applications, including augmented reality (AR), virtual reality (VR), autonomous vehicles, and Internet of Things (IoT) technologies, is transforming digital ecosystems. These technologies demand real-time processing with extremely low response delays to maintain uninterrupted system performance and a high-quality user experience [1]. For instance, in AR/VR applications, the real-time rendering of high-resolution graphics requires rapid data transmission and low-latency computing to avoid motion sickness and visual lag [2]. Autonomous vehicles rely on continuous environmental perception, sensor fusion, and rapid decision making to navigate safely, necessitating millisecond-level inferences to prevent accidents [3]. Similarly, IoT networks generate vast amounts of data from distributed edge devices, thereby requiring efficient real-time processing for services including intelligent urban infrastructure automated industrial systems, and remote medical surveillance [4].

Traditional cloud-computing architectures struggle to meet these stringent requirements owing to high transmission delays, network congestion, and excessive energy consumption associated with long-distance data processing. Consequently, mobile edge computing (MEC) is regarded as a fundamental paradigm that places computing capabilities near end devices, which substantially decreases communication delay and improves energy utilization efficiency [3]. MEC enables edge nodes, such as access points, base stations, and small-scale datacenters, to compute tasks locally rather than offloading them to distant cloud servers. By minimizing data-transmission delays and network congestion, MEC provides low-latency computation, improves bandwidth utilization, and reduces power consumption [5]. MEC also facilitates AI-driven edge intelligence, enabling real-time inference, predictive analytics, and adaptive resource management at the network edges. Despite these advantages, MEC faces critical challenges, especially under highly dynamic scenarios characterized by varying workload requirements and changing network states. Effective management of computation offloading and resource distribution is necessary to maintain system responsiveness and energy efficiency. These challenges become even more complex in large-scale or infrastructure-sparse scenarios [6].

To enhance the flexibility and scalability of MEC networks, unmanned aerial vehicles (UAVs) are increasingly being adopted as mobile edge nodes to provide on-demand computational resources and network coverage in dynamic environments. UAVs are particularly advantageous because of their adaptive mobility, rapid deployment flexibility, and line-of-sight (LoS) transmission characteristics, which make them suitable for temporary high-traffic scenarios, disaster recovery operations, and remote area connectivity [7]. For instance, in a large-scale public event, such as a stadium championship match, network congestion due to thousands of simultaneous users can degrade service quality. Deploying UAV-assisted MEC nodes in such an environment can dynamically offload computational tasks, enhance network capacity, and reduce latency for the real-time services, such as AR/VR overlays, live video streaming, and real-time analytics [8]. Moreover, UAVs can support emergency communication in disaster-stricken regions in which conventional ground-based infrastructure becomes unavailable or damaged [9].

Within UAV-assisted MEC architectures, task offloading serves as a fundamental mechanism for improving quality of service (QoS) through the transfer of computational workloads from user equipment toward edge computing nodes for processing [8]. However, UAVs have limited processing capabilities owing to hardware constraints, and the blind offloading of all tasks can result in network congestion, resource depletion, and increased processing delays [10]. Additionally, available communication resources, including channel bandwidth and transmission energy, remain constrained, resulting in competition among multiple devices attempting to access the computational resources of UAVs [11]. Without efficient computation offloading and resource management strategies, the system may experience inefficient resource utilization, prolonged communication delays, and excessive energy usage [12]. Therefore, an optimized offloading strategy is essential to determine which tasks are processed locally, which need to be offloaded, and how to distribute available resources efficiently to balance the computational load and minimize system overhead.

Beyond computation and communication, caching can play a pivotal role in enhancing MEC performance. In this study, caching does not refer to conventional content caching, such as storing videos, files, or service programs. Instead, we consider task-result caching, where the output of a previously completed deterministic computation task is stored and reused when the same task with identical input is requested again. This approach is particularly meaningful for latency-sensitive MEC applications that generate repetitive computation requests. For example, in AR applications, rendering the same virtual scene or object from a similar viewpoint often requires repeating an identical rendering computation [13,14]. In IoT sensor networks, periodic sensor readings under similar conditions can trigger repeated inference computations, and in autonomous systems, similar sensor inputs, such as recurring traffic scenes, can lead to repeated image-classification computations [14,15]. In such cases, caching the results of frequently requested tasks can significantly reduce both transmission and processing overhead. If a result is already cached, it can be delivered instantly to the user, bypassing the need for recomputation. This not only improves response time but also lowers energy consumption [15]. However, due to limited storage capacity at UAVs and MEC nodes, cache management approaches should be optimally configured to improve the cache hit ratio while operating within resource constraints [12].

**Figure 1:**
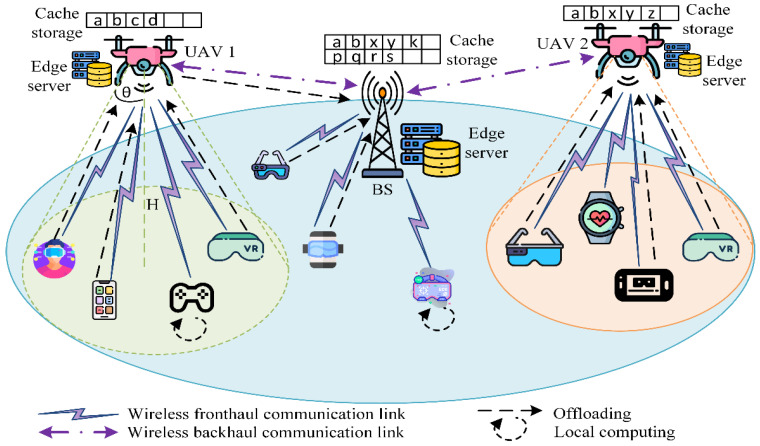
The system model of joint task offloading and resource allocation with data caching in UAV-aided MEC networks.

This study investigates a UAV-aided MEC network, in which several UAVs integrated with edge computing units and cache storages cooperate with a base station (BS) with higher computing and caching capabilities, as shown in Figure 1. The network serves mobile user devices (UDs) on the ground, including smartphones, AR/VR devices, and IoT sensors that generate computational tasks. Each UAV operates at a fixed altitude and provides computing services within its dedicated coverage area where the user density is high. UDs can execute tasks locally or offload them to their assigned UAV, which then determines whether to compute the task locally on UAV or further offload it to the BS for assistance, considering its limited computational capacity.

To reduce latency and energy consumption, tasks must be efficiently offloaded, and resources must be allocated properly. UDs allocate transmission power for offloading, whereas UAVs and BS allocate computing resources for processing. To further reduce computational redundancy, completed task-results can be stored in UAV or BS caches such that repetitive requests can be served directly without reprocessing. This significantly reduces communication and computation overhead and improves system efficiency. However, due to constrained cache storage availability, not all processed tasks can be stored, necessitating an optimized caching policy. Additionally, all tasks have latency constraints; therefore, an effective approach for offloading, resource management, and cache coordination is necessary to guarantee successful task completion.

Although task offloading, resource allocation, and caching have each been widely investigated in UAV-aided MEC networks, most existing studies address only one or two of these components at a time. To the best of our knowledge, among the limited studies that attempt to jointly optimize all three, none combine a UAV-BS collaborative architecture with an entropy-regularized soft actor–critic (SAC)-based deep reinforcement learning (DRL) method for mixed binary-continuous decision making together with a reusable task-result caching mechanism. Existing joint solutions rely on non-learning optimization methods with limited adaptability to dynamic network states, employ DRL algorithms (e.g., DQN, DDQN) that face exploration or scalability limitations in continuous, high-dimensional action spaces, or focus on caching pre-deployed content or services rather than the results of completed computations.

To address this gap, we propose a joint task offloading and resource allocation with data caching (JORC) framework. The considered optimization model is represented as a mixed-integer nonlinear optimization problem owing to the presence of both binary and continuous-valued decision parameters. To solve this problem, we modeled the problem as a Markov decision process (MDP) and employed a DRL method based on the SAC algorithm for optimizing task offloading and resource allocation, along with a hybrid least-frequently used and least-recently used (LFU-LRU) caching mechanism for adaptive data caching. The JORC scheme enables the system to learn adaptive policies that balance latency, energy consumption, and resource utilization under dynamic network conditions.

The primary contributions of this study are summarized below:

- **UAV-BS collaborative architecture:** We designed a UAV-aided MEC framework in which multiple UAVs, each equipped with edge computing and caching capabilities, collaborate with a terrestrial BS to jointly support delay-sensitive and computation-demanding applications, rather than relying on UAV-only or BS-only processing.
- **Joint task offloading and resource allocation with data caching (JORC) scheme:** We developed a JORC framework that integrates SAC-based offloading and resource allocation decisions with a hybrid LFU-LRU task-result caching mechanism within a UAV-BS collaborative architecture. The joint problem is formulated as an MDP and solved via an entropy-regularized, continuous-control DRL approach, enabling adaptive policies that reduce redundant computation while balancing latency, energy, and resource utilization.
- **Adaptive caching mechanism:** We designed a dynamic cache replacement mechanism that jointly considers the access frequency and recency of cached task-result entries. This enables the system to retain computation results with higher reuse probability while adapting to changing request patterns and limited UAV/BS cache capacity.
- **Extensive simulation and performance evaluation:** We conducted comprehensive experiments demonstrating that JORC outperforms existing methods in terms of latency reduction, energy efficiency, successful task completion ratio, and cache hit ratio, confirming the benefit of jointly optimizing offloading and resource allocation, with result caching.

Collectively, these contributions provide a robust, cost-efficient, and adaptive framework for UAV-aided MEC, addressing the key challenges of latency, energy consumption, and resource utilization.

The rest of this paper is structured in the following manner. Section 2 summarizes existing studies and identifies the corresponding research gap. Section 3 presents the system model including the network, task, communication, computation, and caching components. Section 4 defines the considered optimization framework. Section 5 introduces the proposed JORC methodology and associated algorithms. Section 6 presents simulation-based performance analysis and compares the proposed JORC framework with baseline approaches. Section 7 discusses the experimental results, outlines the assumptions and limitations of the proposed framework, and identifies promising directions for future research. Lastly, Section 8 presents the concluding remarks and highlights potential future research directions.

## 2. Related Works

The increasing demand for latency-sensitive and computation-intensive applications has driven extensive research in UAV-aided MEC networks. Prior studies have explored task offloading, resource allocation, and caching from different perspectives, but significant challenges remain in effectively combining these components under UAV-BS collaborative environments. This section reviews the existing studies, highlights key advancements, and identifies the gaps that our proposed JORC framework aims to address.

*Computation offloading in MEC*: Task offloading is a key component of MEC systems, enabling devices with limited computational power to execute complex tasks by offloading them onto edge or cloud servers. Various studies have explored heuristic, game-theoretic, and deep-learning-based approaches for optimizing offloading decisions. In [9], the authors proposed the JDACO framework in post-disaster UAV-assisted IoT environments, leveraging a VD3QN-based multi-agent RL model to minimize latency and energy consumption. Ref. [16] introduced an AI-driven aerial MEC framework for jointly optimizing offloading, device association, and UAV trajectory planning using a knapsack-based task selection strategy. A layered MEC architecture with edge UAVs and a link UAV was also proposed in paper [17], employing the AGSP algorithm to optimize offloading and UAV deployment. More recently, cooperative multi-UAV MEC studies have jointly optimized task offloading, computation-resource allocation, and trajectory design to reduce latency and energy consumption [18], while service-capacity-oriented UAV-MEC frameworks have emphasized deadline-constrained task completion under limited UAV resources [19]. While these works advance offloading strategies, they generally do not exploit task-result caching for avoiding repeated computation in UAV-BS collaborative MEC systems.

*Offloading and resource allocation in MEC*: Efficient management of resources such as bandwidth and CPU frequency is critical for ensuring low latency and high throughput in UAV-aided MEC environments. Several studies have integrated offloading decisions with resource allocation, but lack caching mechanisms, which can significantly reduce computational redundancy. In [20], the authors proposed a cloud-edge UAV framework where a hybrid SS-DQN algorithm coordinates task offloading with resource allocation. Ref. [3] introduced a hybrid DRL-greedy algorithm for vehicular networks to allocate bandwidth and CPU resources based on task demands and mobility. In hierarchical UAV systems, a MAPPO-based reinforcement learning model is applied in [21] for optimizing offloading ratios and multi-dimensional resource allocation. In [22], the authors combined GRU-attention mechanisms in a multi-agent setup for optimizing offloading while allocating computing and communication resources. Recent DRL-based UAV-MEC studies have also used PPO or DQN-style learning to jointly optimize offloading, trajectory, bandwidth, and computation-resource allocation in dynamic aerial edge environments [23,24]. Most recently, the authors in [25] proposed a multi-agent TRPO-based hierarchical offloading framework for large-scale UAV networks. However, these approaches mainly focus on offloading and resource-control decisions and do not integrate the completed task-result reuse through adaptive cache replacement.

*Offloading and caching in MEC*: Caching can significantly reduce redundant computations and improve system responsiveness by storing frequently accessed data. Several studies have integrated offloading and caching, but they overlook resource allocation optimization. A federated DRL-based model is proposed in [26] for UAV-assisted vehicular networks, enabling dynamic caching and offloading with local model training. In [13], the authors applied DDPG for offloading decisions together with cooperative caching scheme in multi-region MEC environments to enhance efficiency. A hierarchical DRL model is also developed in [27] to jointly manage caching and offloading in edge-cloud systems, utilizing DQN for service caching and SAC for offloading decisions. Cooperative caching and offloading strategies have also been proposed in [14] for mobile augmented reality and vehicular networks, optimizing cache placement and offloading via dynamic programming and artificial bee colony algorithms. Recent UAV-assisted vehicular networks and Internet of vehicles (IoV) studies have further combined task offloading with fuzzy caching, service caching, or content caching using hierarchical DRL, A3C, or DDPG-based methods [28,29,30]. The authors in [31] combined multi-agent DRL with federated-learning-based caching for UAV-assisted IoV. In addition, multi-UAV and UAV-BS offshore MEC systems have considered service-instance caching, user-preference-oriented service caching, and service retrieval from BS/cloud nodes to reduce service delay and backhaul dependence [32,33,34]. Nevertheless, these studies mainly cache contents, service programs, or service instances rather than the completed computation results.

*Offloading, resource allocation, and caching*: A few studies have attempted joint optimization of offloading, resource assignment, and caching but failed to consider UAV-BS collaboration, limiting their adaptability to UAV-aided MEC networks. In [15], the authors proposed a collaborative framework where mobile users offload tasks to MEC servers while cached results are reused to reduce delay and energy. It jointly determines offloading, caching, and allocation of CPU frequency and transmission power. A meta-reinforcement learning approach had also been introduced in [35] to simultaneously optimize offloading, caching, and resource allocation under dynamic user preferences. The approach explored a two-tier network architecture, comprising edge nodes and mobile devices. It employed an RNN-based sequence-to-sequence (seq2seq) network to model the policy and value functions, transforming the problem of solving multiple MDPs into a task execution sequence prediction. A three-tier MEC architecture is also developed in [36], applying a DDQN-based model to coordinate offloading, service caching, and resource allocation across user devices, edge servers, and the cloud layer. In [37], the authors jointly optimized offloading, cache resource assignment, and routing in a satellite-UAV space-air-ground network using program caching, and the authors in [38] jointly optimized offloading, resource allocation, and service caching for multi-UAV MEC using a dual-timescale TD3 algorithm. Furthermore, service experience-oriented optimization frameworks were proposed in [39] to adjust caching placement, UAV trajectories, and resource distribution to maximize fairness and minimize latency. Both refs. [36,39] focused on caching, but they investigated service caching rather than result caching. Recent UAV-assisted MEC studies have also optimized content caching, computation offloading, and channel allocation under multi-task parallel execution [40], and jointly considered service caching, task offloading, CPU allocation, UAV scheduling, and service delivery for latency-critical UAV networks [41]. However, these approaches still focus on content/service/program caching and do not directly reuse the completed task-results for repeated or similar computation requests.

Beyond learning-based methods, conventional approaches have also been used to address joint resource allocation and caching problems in MEC. Some studies adopt iterative or metaheuristic techniques, such as the non-DRL iterative framework in [15] and the dynamic programming and artificial-bee-colony-based approach in [14]. Others formulate the problem as a mixed-integer nonlinear optimization (MINLP) program, solved via convex relaxation, alternating optimization, or Lagrangian decomposition. For instance, Yang et al. [42] addressed energy minimization for binary computation offloading in an IRS-aided MEC system using a penalty-based conventional optimization approach. Similarly, Chen et al. [43] jointly optimized hybrid beamforming and content placement in an RIS-aided edge-caching system using an alternating optimization technique. These approaches provide strong optimality guarantees for a given network realization, but generally require the optimization problem to be resolved whenever channel conditions, task arrivals, or cache states change, which limits their scalability in continuously evolving UAV-aided MEC environments with time-varying task arrivals and cache occupancy.

While previous studies have made significant contributions in task offloading, resource allocation, and caching, they fail to integrate all three aspects dynamically under UAV-BS collaboration. Most studies have optimized only two components, resulting in inefficient resource utilization. Studies without UAV-BS collaboration fail to efficiently manage task distribution and resource sharing. Existing DRL-based solutions use DQN, DDQN, A3C, DDPG, or PPO-style models, which may face limitations in exploration stability, convergence behavior, or scalability when handling continuous offloading and resource allocation decisions in dynamic UAV environments. Most caching techniques focus on content or service caching, whereas our framework optimizes task-result caching to reduce redundant computations.

To address these gaps, we propose the JORC framework, that jointly coordinates task offloading and resource allocation decisions using a SAC-based DRL approach and integrates a hybrid LFU-LRU caching strategy to reduce computational redundancy. Table 1 summarizes representative studies on task offloading, resource allocation, and caching in UAV-aided MEC networks, not only comparing their coverage of each component, caching type, UAV-BS collaboration, and solution method but also highlighting how each differs from the proposed JORC framework. Unlike [15], which uses a non-DRL optimization method, and [35], which employs a meta-reinforcement learning approach with an RNN-based seq2seq model, JORC leverages SAC’s entropy regularization and off-policy learning, which offer faster convergence and better exploration–exploitation balance in continuous action spaces. Unlike [36,39], which focus on service caching (i.e., caching pre-deployed application services), JORC targets task-result caching, where the computed outputs of specific task instances are stored and reused to directly avoid redundant computation and transmission. Furthermore, none of the existing approaches [15,35,36,39] operate within a UAV-BS collaborative architecture where UAVs with limited capacity dynamically offload tasks to a more powerful BS, which is a key architectural distinction of JORC. By combining SAC-driven offloading and resource allocation control, UAV-BS cooperation, and adaptive result caching, JORC provides a scalable and effective solution for UAV-aided MEC under dynamic network conditions.

## 3. System Model

This section provides a thorough description of the system model, covering the network, communication, task, offloading, computation, and caching components. Table 2 summarizes the main notations used throughout the paper.

### 3.1. Network Model

The system under consideration consists of a single BS and multiple UAVs, where both the BS and UAVs are equipped with edge servers to support task execution. Formally, the BS is represented as b and the set of UAVs is represented as *m* ∈ *M* = {1, 2, 3, …, *M*}. Additionally, the system includes *N* user devices (UDs), such as smartphones, AR, VR, and IoT devices, each requiring computational resources for task execution. The set of UDs is represented as *n* ∈ *N* = {1, 2, 3, …, *N*}. The spatial distribution of BS, UAVs and UDs is represented using a 3D Cartesian coordinate system. Since the BS is located on the ground, its position is defined as $q_{b}=(0,\;0)$. For UAV *m*, the horizontal position is defined by $q_{m}=(x_{m},y_{m})$, where *m* ∈ *M*. Each UAV is equipped with a directional antenna with beamwidth *θ* and operates at a fixed altitude *H*. The height of user devices is negligible in comparison to the altitude of the UAVs. The horizontal coordinate of user device *n* is defined by $q_{n}=(x_{n},y_{n})$, where *n* ∈ *N*. A quasi-static scenario is considered where UAVs and user devices remain stationary within a task offloading period, although their positions may vary across different periods [44].

### 3.2. Communication Model

UAVs establish wireless backhaul connections with a BS, and separate frequency bands are allocated for fronthaul and backhaul communications to reduce signal interference [44]. The fronthaul links connect UDs to the UAVs, while the backhaul links connect the UAVs to the BS. For ease of modeling, the backhaul capacity from the BS to each UAV was considered as $R_{m,b}$. To mitigate co-channel interference over fronthaul transmissions, OFDMA (i.e., orthogonal frequency-division multiple access) is adopted [15]. Considering the elevation of UAVs, we consider the wireless connections between UAVs and devices to be LoS. Because of the mobility consideration as a quasi-static scenario, we may ignore the Doppler Effect. Therefore, the channel gain between device *n* and UAV *m* is modeled as

(1) $$h_{n,m}=\frac{h_{0}}{H^{2}+d_{n,m}^{2}},\;\;$$

where $h_{0}$ is the reference channel gain at 1 *m*, and $d_{n,m}=\left\|q_{m}-q_{n}\right\|=\sqrt{{\left(x_{m}-x_{n}\right)}^{2}+{\left(y_{m}-y_{n}\right)}^{2}}$, which is the horizontal distance between the UAV *m* and UD *n*.

The service radius of UAV *m* is defined by *H* tan *θ,* where *H* denotes the UAV’s altitude and *θ* denotes the antenna beamwidth angle. Therefore, for a UD *n* to offload its task to UAV *m*, the horizontal distance $d_{n,m}$ must not exceed *H* tan *θ* (i.e., $d_{n,m}$ ≤ *H* tan *θ*). Accordingly, the uplink transmission rate of device *n* to UAV *m* is given by

(2) $$R_{n,m}=B_{n}\log_{2}(1+\frac{P_{n}h_{n,m}}{B_{n}N_{0}}),\;\;$$

where $P_{n}$ represents the power transmitted by UD *n*, $N_{0}$ denotes the spectral density of noise at the receiver, $B_{n}\;$ represents the bandwidth assigned to UD *n.*

### 3.3. Task Model

Each UD generates computational tasks that must be either executed locally or offloaded to a UAV or the BS. A computational task of UD *n* at time *t* is characterized by a tuple $\left(T_{n},L_{n}^{max}\right)$, where $T_{n}$ denotes the task information, and $L_{n}^{max}$ denotes the maximum tolerable latency for that task. Task information $T_{n}$ is represented as $\left(D_{n}^{in},W_{n},D_{n}^{out}\right)$; where $D_{n}^{in}$ denotes the task size, $W_{n}$ denotes the CPU cycles required (computing workload) to compute the task, and $D_{n}^{out}$ represents the size of the result after the completion of the task. To ensure QoS, each task must be executed within its latency deadline.

### 3.4. Offloading Model

A UD *n* can send its computational task to the MEC server deployed at UAV *m* or the BS. The offloading decision variable ${X=\{x}_{n}^{UD},x_{n,m}^{UAV},x_{n}^{BS}\}\in \left\{0,1\right\}$, where  n∈N and m∈M. $x_{n}^{UD}=1$ means that the task is executed locally at UD n, $x_{n,m}^{UAV}=1$ indicates the task is assigned to UAV m, and $x_{n}^{BS}=1$ indicates that the task is further offloaded to BS for execution, otherwise 0. To ensure mutual exclusivity in task execution, only one offloading decision is permitted per task, satisfying the following constraint:

(3) $$x_{n}^{UD}+\sum_{m\in M}x_{n,m}^{UAV}+x_{n}^{BS}=1.\;\;$$

If the task is offloaded, the system must account for both uplink and downlink transmission latency and energy consumption.

*Uplink transmission latency (UD to UAV)*: The uplink transmission latency occurs when UD *n* transmits the task input data $D_{n}^{in}$ to UAV *m*. For this offloading case, the latency is expressed as

(4) $$L_{n,m}^{off}=\frac{D_{n}^{in}}{R_{n,m}},\;$$

where $R_{n,m}$ is the uplink data rate from UD *n* to UAV *m*; otherwise, the latency is zero.

*Uplink transmission energy consumption (UD to UAV)*: the corresponding uplink energy consumed for this transmission is expressed as

(5) $$E_{n,m}^{off}=\frac{{P_{n,m}D}_{n}^{in}}{R_{n,m}},\;$$

where $P_{n,m}$ is the transmit power of UD n.

*Uplink transmission latency (UAV to BS)*: if UAV *m* further forwards the task to the BS for computation, then the uplink transmission latency is expressed as

(6) $$L_{n,m,b}^{off}=\frac{D_{n}^{in}}{R_{m,b}},\;$$

where $R_{m,b}$ represents the backhaul capacity.

*Uplink transmission energy consumption (UAV to BS)*: The energy required for this UAV to BS transmission is computed as

(7) $$E_{n,m,b}^{off}=\frac{{P_{m,b}D}_{n}^{in}}{R_{m,b}},\;$$

where $P_{m,b}$ is the uplink transmit power of UAV *m*. If the task is executed locally on UD, no uplink transmission is required; therefore, the corresponding delays and energy terms are zero.

*Downlink transmission latency and energy consumption:* due to the relatively small size of task-results and the MEC to UD downlink rates being typically high, the downlink transmission latency and energy consumption are considered negligible and, thus, are omitted from the analysis [14,15].

### 3.5. Computation Model

Computation task $T_{n}$ of UD n can be either computed locally on UD *n*, or remotely at UAV *m*, or the BS.

*Computing locally on UDs*: When the task $T_{n}$ is processed by UD *n*, then the computation latency is given by

(8) $$L_{n}^{UD}=\frac{W_{n}}{f_{n}},\;$$

where $W_{n}$ denotes the computing workload (required CPU cycles) of task $T_{n}$ and $f_{n}$ denotes the local computing ability in CPU cycles per second of UD *n*. The corresponding energy consumption is expressed as

(9) $$E_{n}^{UD}=k\;f_{n}^{2}\;W_{n},\;$$

where k is the energy-conversion coefficient determined by the processor chip design.

*Computing at UAVs*: When the task $T_{n}$ is processed at UAV server m, then the latency is calculated as

(10) $$L_{n}^{UAV}=\frac{W_{n}}{s_{m,n}F_{u}},\;\;$$

where $W_{n}$ represents the computing workload (required CPU cycles) of task $T_{n}$, $F_{u}$ represents the total computing ability in CPU cycles per second of UAV *m*, and $s_{m,n}\in \left[0,1\right]$ represents the percentage of UAV *m*’s computation resource assigned to UD *n*. The corresponding energy consumption is calculated as

(11) $$E_{n}^{UAV}=k{\left(s_{m,n}F_{u}\right)}^{2}W_{n},\;$$

where k is the energy-conversion coefficient determined by the processor chip design.

*Computing at BS*: When the task $T_{n}$ is processed at the BS, then the latency is expressed as

(12) $$L_{n}^{BS}=\frac{W_{n}}{s_{b,n}F_{b}},\;\;$$

where $W_{n}$ denotes the computing workload (required CPU cycles) of task $T_{n}$, $F_{b}$ denotes the total computing ability (CPU cycles per second) of the BS, and $s_{b,n}\in \left[0,1\right]$ represents the percentage of the BS’s computation resource assigned to UD *n*. The corresponding energy consumption is calculated as

(13) $$E_{n}^{BS}=k{\left(s_{b,n}F_{b}\right)}^{2}W_{n},$$

where k is the energy-conversion coefficient determined by the processor chip design.

### 3.6. Caching Model

In the proposed system, caching refers to the process through which a MEC server (either a UAV or the BS) stores the output of a completed task together with its associated data. When a user device (UD) later requests to execute the same task with an identical input, the MEC server compares the new request with the stored entries and identifies it as a cache hit. In this case, the UD does not need to offload the task again, and the MEC server avoids recomputation. Instead, the previously stored result is returned directly to the UD. This approach reduces transmission delay, computation delay, and overall energy consumption.

The proposed framework automatically caches the result of each newly executed offloaded task. Since MEC servers have limited storage capacity, not all task-results can be stored simultaneously. To satisfy the storage constraint, the total amount of data cached at MEC server *i* must remain within its maximum storage limit $S_{i}^{max}$. That is,

(14) $$\sum_{n\in N}x_{n}^{i}.D_{n}^{out}\leq S_{i}^{max},\;\;\;\;\forall i\in M\cup \{b\},\;$$

where $S_{i}^{max}$ denotes the available storage limit at MEC server *i*. This equation ensures that the total cached data at MEC server *i* does not exceed its storage limit.

In our proposed framework, the cache lookup is performed based on a unique task identifier. When the same computational task with identical input is requested again, the system recognizes it as a cache hit and retrieves the stored result directly, bypassing recomputation [15]. This assumption is well-suited for applications that generate deterministic and repetitive computational requests, such as fixed AR scene rendering, repeated sensor data inference in IoT networks, or periodic image-classification tasks in autonomous systems [14,15].

## 4. Problem Formulation

This study aims to minimize the overall cost of a UAV-aided MEC system. Cost is modeled as a weighted combination of latency and energy consumption. Because the units of latency and energy are different, we used the normalized latency and energy values.

In the proposed system, a cache hit avoids task offloading and computation, allowing the result to be delivered directly to the user. Only cache query latency and energy are involved, which are negligible compared to offloading and computation costs. In contrast, a cache miss requires full computation and communication latency and energy to be considered.

The total latency associated with a single task is calculated as follows:

(15) $$L_{n}=\;x_{n}^{UD}\left(L_{n}^{UD}\right)+x_{n,m}^{UAV}\left(L_{n,m}^{off}+L_{n}^{UAV}\right)+x_{n}^{BS}\left(L_{n,m}^{off}+L_{n,m,b}^{off}+L_{n}^{BS}\right).\;$$

Similarly, the total energy required for a single task is expressed as follows:

(16) $$E_{n}=\;x_{n}^{UD}\left(E_{n}^{UD}\right)+x_{n,m}^{UAV}\left(E_{n,m}^{off}+E_{n}^{UAV}\right)+x_{n}^{BS}(E_{n,m}^{off}+E_{n,m,b}^{off}+E_{n}^{BS})\;.\;\;$$

Hence, the cost for a single user can be defined as

(17) $$C_{n}=\gamma_{n}^{L}.L_{n}^{\prime }+\gamma_{n}^{E}.E_{n}^{\prime },\;\;$$

where $L_{n}^{\prime }$ and $E_{n}^{\prime }$ denote the normalized values of latency and energy consumption, respectively, and $L_{n}^{\prime }=L_{n}/L_{n}^{max}$ and $E_{n}^{\prime }=E_{n}/E_{n}^{max}$. $L_{n}^{max}$ and $E_{n}^{max}$ are the maximum values of $L_{n}$ and $E_{n}$ respectively. $\gamma_{n}^{L}$ and $\gamma_{n}^{E}$ are the weight factors of latency and energy consumption, respectively. The range of these weighted factors are $0\leq \gamma_{n}^{L}\leq 1$ and $0\leq \gamma_{n}^{E}\leq 1$, respectively, where $\gamma_{n}^{L}\;+\;\gamma_{n}^{E}$ = 1.

Therefore, the overall cost for all users can be defined as

(18) $$C=\sum_{n=1}^{N}C_{n}.\;\;$$

Our goal is to minimize the overall cost of the system. Hence, the optimization problem can be expressed as follows:

(19a) $$\underset{X,P,F}{\min}C(X,P,F)\;$$

subject to:

(19b) $${X=\{x}_{n}^{UD},x_{n,m}^{UAV},x_{n}^{BS}\}\in \left\{0,1\right\},\forall n\in N,\forall m\in M$$

(19c) $$x_{n}^{UD}+\sum_{m\in M}x_{n,m}^{UAV}+x_{n}^{BS}=1,\;\;\forall n\in N,\forall m\in M\;\;$$

(19d) $$L_{n}\leq L_{n}^{max}$$

(19e) $$0<P\leq P^{max}$$

(19f) $$\sum_{n=1}^{N}s_{i,n}F_{i}\leq F_{i},\forall i\in M\cup \{b\}.$$

where *X* represents the set of offloading decision variables, *P* denotes the transmit power allocation variable, and *F* denotes the variable of computing frequency allocated by the MEC servers (UAVs and the BS). Constraint (19b) defines the binary offloading decisions, whereas (19c) ensures that each task is computed at exactly one location (UD, UAV, or BS). Constraint (19d) requires the completion latency of each task to remain within its maximum tolerable threshold. Constraint (19e) guarantees that the allocated transmit power is strictly positive and does not exceed the maximum allowable limit. Finally, constraint (19f) restricts the total CPU frequency allocation of all tasks within the computing capacity of each MEC server.

## 5. Joint Task Offloading and Resource Allocation with Data Caching (JORC) Scheme

**Figure 2:**
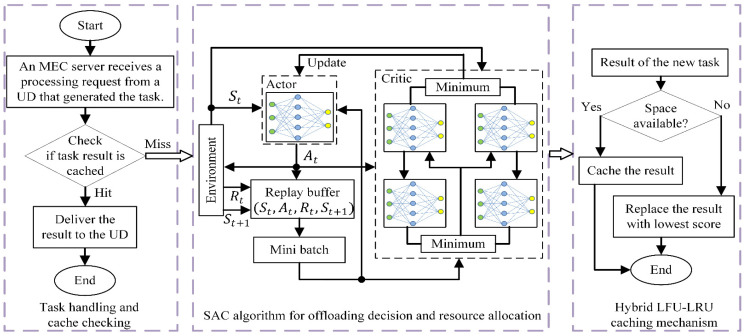
Architecture of our proposed JORC scheme consisting of (i) task handling and cache checking, (ii) SAC algorithm for offloading decision and resource allocation, and (iii) hybrid LFU-LRU caching mechanism (All the operations in Figure 2 are carried out by the MEC servers (i.e., UAVs and BS)).

This section explains the design and operational flow of the proposed JORC algorithm, which aims to reduce the overall cost of a UAV-BS collaborative system. JORC integrates a SAC-based DRL mechanism for offloading decisions and resource allocation, alongside a hybrid LFU-LRU caching strategy to improve storage efficiency. The architecture of the proposed JORC scheme is illustrated in Figure 2.

The JORC framework operates sequentially to optimize real-time decision making in UAV-BS collaborative networks. When a UD generates a task, an offloading request is first sent to the UAV or BS. The UAV or BS then checks whether the requested task-results have been cached. If a cache hit occurs, the result is retrieved and delivered immediately to the UD, thereby eliminating repeated computation. For a cache miss, the SAC-based decision making process determines the optimal execution location and allocates computational and communication resources. Once the task has been executed, the result is stored in the cache using the LFU-LRU policy, making it available for immediate reuse in future requests.

### 5.1. SAC Algorithm for Joint Offloading and Resource Management

Although the communication model adopts a quasi-static large-scale channel model, the overall UAV-aided MEC system remains dynamic because user locations, task arrivals, available computing resources, and workload distribution vary over time. Consequently, the JORC framework must repeatedly determine task offloading and resource allocation decisions as these conditions evolve. The underlying offloading and resource allocation problem is formulated as a non-convex mixed-integer nonlinear optimization problem. Solving it online repeatedly as the system state changes can be computationally demanding, particularly under the strict per-task latency constraints considered in this study. Therefore, the proposed SAC-based DRL framework learns an adaptive decision policy offline, which can subsequently be applied efficiently online without repeatedly solving the optimization problem.

The choice of SAC over other DRL algorithms is motivated by the specific characteristics of the joint offloading–resource management task in UAV-aided MEC. First, the action space of JORC is continuous and high-dimensional, comprising offloading decisions, transmission power allocation, and CPU frequency allocation simultaneously. SAC is inherently designed for continuous action spaces through its stochastic actor formulation, whereas algorithms such as DQN and DDQN are restricted to discrete action spaces and would require action discretization that introduces approximation errors. Second, relative to on-policy methods such as PPO, SAC’s off-policy learning with a replay buffer significantly improves sample efficiency, which is critical in dynamic MEC environments where poor decisions carry real costs. Third, compared to TD3, which also handles continuous actions, SAC’s entropy regularization explicitly encourages exploration by maintaining policy stochasticity, preventing premature convergence to suboptimal offloading strategies in early training stages. This is particularly valuable in UAV-aided MEC, where the joint state space (user positions, channel conditions, resource availability, and cache state) is large and constantly evolving. The empirical convergence results in Section 6.4.1 further validate this choice, showing that SAC achieves stable convergence approximately 330 episodes faster than PPO and 680 episodes faster than A3C.

#### 5.1.1. MDP Representation

The joint offloading and resource-management problem in the UAV-aided MEC system is represented as an MDP to support adaptive decision making under dynamic network conditions. An MDP is specified by a tuple (*S*, *A*, P, *R*, *γ*), where *S* is the state space, *A* is the action space, P is the state-transition probability, *R* is the reward function, and *γ* is the discount factor that balances immediate and future rewards.

*State space*$\left(S_{t}\right)$: The state captures the current system condition, including:

(20) $$S_{t}=\left\{S_{t}^{UD},S_{t}^{UAV},S_{t}^{BS},S_{t}^{network}\right\},$$

where $S_{t}^{UD}$ represents the user information (including task information $T_{n}$, maximum tolerable latency $L_{n}^{max}$, energy status $E_{n}^{UD}$ and position of the UD $\left(x_{n},y_{n}\right)$), $S_{t}^{UAV}$ denotes the UAV state (available CPU resources $F_{m}$, current workload, and position of the UAV $\left(x_{m},y_{m}\right)$), $S_{t}^{BS}$ denotes the state of BS (available computing capacity $F_{b}$), and $S_{t}^{network}$ represents the network state (channel gain $h_{n,m}$ between user *n* and UAV *m*, and network congestion status).

*Action space*$\left(A_{t}\right)$: This space defines the control variables used for offloading decisions and resource allocation. At each time step *t*, action $A_{t}$ is represented as

(21) $$A_{t}=\left\{X,P,F\right\}.$$

Because the offloading decision X in (3), (19b), and (19c) is binary and mutually exclusive, whereas the SAC actor produces continuous outputs, a projection step is used to obtain a feasible offloading decision. For each user n, the actor outputs continuous-valued preference scores for each candidate execution location, $z_{n}=\left[z_{n}^{UD},z_{n}^{UAV},z_{n}^{BS}\right]$. The execution location is selected as

(21a) $$i_{n}^{*}=arg\underset{\mathrm{i\epsilon}\{\mathrm{UD},\mathrm{UAV},\mathrm{BS}\}}{\max}z_{n}^{i},$$

and the corresponding binary offloading decision is obtained as

(21b) $$x_{n}^{i}=\{\begin{array}{c} 1,\;\;\;\;\;i=i_{n}^{*},\;\;\;\;\;\;\;\;\;\\ 0,\;\;\;\;\;otherwise, \end{array}$$

thereby satisfying the binary and mutual exclusivity constraints in (19c). The remaining action components, namely transmit power and CPU frequency allocation, remain continuous.

*Reward*${(R}_{t})$: SAC aims to maximize the accumulated reward, whereas our target is to minimize cost. Therefore, the reward is set as the negative system cost, as follows:

(22) $$R_{t}=-C\left(t\right).$$

*Transition probability*${(P}_{t})$*:* The state transition probability $P_{t}(S_{t+1}|S_{t},A_{t})$ describes the probability of reaching state $S_{t+1}$ from the current state $S_{t}$, when action $A_{t}$ is chosen. In this work, the transition probability is not assumed to be known in closed form and is neither explicitly modeled nor estimated. Instead, the proposed SAC algorithm learns directly from the sampled transitions $\left(S_{t},A_{t},R_{t},S_{t+1}\right)$ generated through interaction with the environment, without requiring prior knowledge of the transition dynamics.

#### 5.1.2. Soft Actor–Critic (SAC) Algorithm

In the SAC algorithm, the expected reward and entropy are included in the objective function to maximize. The expected entropy ensures stability and exploration through random actions. The main goal is to learn an optimal policy $\pi^{*}$ that maximizes both the expected reward and entropy, defined as

(23) $$\pi^{*}=\arg\underset{\pi }{\max}\sum_{t}E\left[R_{t}\left(S_{t},A_{t}\right)+\alpha H\left(\pi \left(\cdot |S_{t}\right)\right)\right],$$

where E[·] denotes the expectation of a function, $H\left(\pi \left(\cdot |S_{t}\right)\right)$ represents entropy of policy π in state $S_{t}$, and α represents the temperature parameter that defines the relative significance of entropy and controls the stochasticity of the policy. The temperature parameter should be dynamic because the policy gradually improves with experience; therefore, less exploration is needed when there is a clear distinction between poor and optimal actions. The dynamic entropy adjustment in SAC was first proposed in [45]. This is crucial, as it allows the policy to adapt dynamically, ensuring sufficient exploration when the best action is ambiguous, while becoming less stochastic in states where the preferred action is evident. This dynamic adjustment improved the performance and stability of the learning algorithm. We considered this dynamic temperature adjustment when designing the SAC algorithm.

Algorithm 1 describes the training procedure of the proposed SAC model. The SAC algorithm has two critic networks, $Q_{1}$ and $Q_{2}$ with parameters $\theta_{Q_{1}}$ and $\theta_{Q_{1}}$, two target critic networks, $Q_{1}^{\prime }$ and $Q_{2}^{\prime }$ with parameters $\theta_{Q_{1}^{\prime }}$ and $\theta_{Q_{2}^{\prime }}$, and actor-network π with parameter $\theta_{\pi }$. The agent observes the environmental state $S_{t}$, then selects an action $A_{t}$ according to the current policy π. After executing the action, the agent receives a reward $R_{t}\left(S_{t},A_{t}\right)$ and transitions to the next state $S_{t+1}$, and then stores the transition tuple $\left(S_{t},A_{t},\;R_{t},\;S_{t+1}\right)$ in the replay buffer *D*.
**Algorithm 1.** SAC algorithm for task offloading and resource-management decisions.**Input:** Maximum number of episodes, maximum number of steps in each episode, environment state information $S_{t}$, reward function $R_{t}\left(S_{t},A_{t}\right)$, learning rates λ, discount factor γ, initial temperature parameter α, and constant ε. **Output:** Optimal X, P, and F. 1:   Initialize actor network $\pi (A|S;\theta_{\pi })$ with parameters $\theta_{\pi }$ 2:   Initialize two critic networks $Q_{1}(S,A;\theta_{Q_{1}})$ and $Q_{2}(S,A;\theta_{Q_{2}})$ with parameters $\theta_{Q_{1}}$ and $\theta_{Q_{2}}$, and two target critic networks $Q_{1}^{\prime }$ and $Q_{2}^{\prime }$ with parameters $\theta_{Q_{1}^{\prime }}\leftarrow \theta_{Q_{1}}$, $\theta_{Q_{2}^{\prime }}\leftarrow \theta_{Q_{2}}$ 3:   Initialize temperature parameter *α*, replay buffer *D*, target entropy *H* 4:   **for** each episode **do** 5:     Reinitialize environment and receive initial state $S_{0}$ 6:     **for** each time step in an episode **do** 7:       Choose action $A_{t}\;$ based on current policy and execute it 8:       Obtain a reward $R_{t}\left(S_{t},A_{t}\right)$ and a next state $S_{t+1}\sim P_{tn}(S_{t+1}|S_{t},A_{t})$ 9:       Append transition tuple $\left(S_{t},A_{t},\;R_{t},\;S_{t+1}\right)$ to the reply buffer *D* 10:     Draw a mini batch of samples $\left(S_{t},A_{t},\;R_{t},\;S_{t+1}\right)$ from *D*
11:     update critic parameters based on (27)        $\;\theta_{Q_{i}}\leftarrow \theta_{Q_{i}}-\lambda_{Q}\nabla_{\theta_{Q_{i}}}J_{Q}\left(\theta_{Q_{i}}\right)$ for i∈{1, 2} 12:     update actor parameters based on (30)         $\;\theta_{\pi }\leftarrow \theta_{\pi }-\lambda_{\pi }\nabla_{\theta_{\pi }}J_{\pi }\left(\theta_{\pi }\right)$
13:     update temperature parameter by minimizing (31)        $\;\alpha \leftarrow \alpha -\lambda_{\alpha }\nabla_{\alpha }J\left(\alpha \right)$
14:     update target critic network parameters by (32)        $\;\theta_{Q_{i}^{\prime }}\leftarrow \varpi \theta_{Q_{i}}+\left(1-\varpi \right)\theta_{Q_{i}^{\prime }}$ for i∈{1, 2} 15:    **end for** 16: **end for**

Policy evaluation, policy improvement, and automatic temperature parameter adjustment occurred during the SAC training phase.

To evaluate the soft policy, first we need to calculate soft Q values, which are computed as

(24) $$Q\left(S_{t},A_{t}\right)=R_{t}\left(S_{t},A_{t}\right)+\gamma E_{S_{t+1}\sim P_{tn}}\left[V\left(S_{t+1}\right)\right],\;\;$$

where γ denotes the discount factor and $V\left(S_{t+1}\right)$ represent the soft value function for state $S_{t+1}$, which can be defined as

(25) $$V\left(S_{t}\right)=E_{A_{t}\sim \pi }[Q\left(S_{t},A_{t}\right)-\alpha \log\pi \left(A_{t}\right|S_{t})].\;$$

The parameter θ of policy evaluation network (critic) Q is updated to minimize the loss function represented as

(26) $$J_{Q}\left(\theta \right)=E_{(S_{t},A_{t})\sim D}\left[\frac{1}{2}{\left(Q\left(S_{t},A_{t}\right)-\left(R_{t}\left(S_{t},A_{t}\right)+\gamma E_{S_{t+1}\sim P_{tn}}\left[V_{\theta_{Q^{\prime }}}\left(S_{t+1}\right)\right]\right)\right)}^{2}\right],\;\;\;$$

where the soft state value function is calculated using Equation (24) with target parameters $\theta_{Q^{\prime }}$. We use gradient descent with mini-batch samples to update θ:

(27) $$\nabla_{\theta }J_{Q}\left(\theta \right)=\nabla_{\theta }Q_{\theta }\left(S_{t},A_{t}\right)\left(Q_{\theta }\left(S_{t},A_{t}\right)-\left(R_{t}\left(S_{t},A_{t}\right)+\gamma \left(Q_{\theta_{Q^{\prime }}}\left(S_{t+1},A_{t+1}\right)-\log\left(\pi (A_{t+1}|S_{t+1}\right)\right)\right)\right),\;$$

Next, in the policy improvement stage, parameters $\theta_{\pi }$ of policy network (actor) π is updated by minimizing the expected KL-divergence:

(28) $$J_{\pi }\left(\theta_{\pi }\right)=E_{(S_{t},A_{t})\sim D}\left[E_{A_{t}\sim \pi }\left[\alpha \log\pi \left(A_{t}\right|S_{t})-Q\left(S_{t},A_{t}\right)\right]\right].\;\;\;$$

The details of obtaining (28) using the KL divergence are provided in [46]. To calculate the gradient descent of (27), we need to reparametrize $\;A_{t}$, which helps to reduce the gradient estimation variance during policy optimization. We begin by selecting a random sample $\psi_{t}$ from a predetermined distribution (e.g., standard normal distribution). Next, we derive $A_{t}$ as

(29) $$A_{t}=\mu_{t}+\psi_{t}⊙\varepsilon_{t},\;\;\;$$

where $\mu_{t}$ represents the mean of the policy, $\varepsilon_{t}$ denotes the standard deviation of policy π, and ⊙ signifies the Hadamard product. Then, the gradient update is calculated as

(30) $$\;\;\nabla_{\theta_{\pi }}J_{\pi }\left(\theta_{\pi }\right)=\nabla_{\theta_{\pi }}\alpha \log\pi \left(A_{t}\right|S_{t})+\left(\nabla_{A_{t}}\alpha \log\pi \left(A_{t}\right|S_{t})-\nabla_{A_{t}}Q\left(S_{t},A_{t}\right)\right)\nabla_{\theta_{\pi }}A_{t}.\;$$

We use two critic networks, each parameterized by $\theta_{Q_{i}},\;i\;\epsilon \;\{1,\;2\}$, to mitigate positive bias during policy improvement, which can degrade algorithm performance. By independently training the networks to optimize $J_{Q}\left(\theta_{Q_{i}}\right)$ and using the minimum Q-value in (27) and (30), the clipped double-Q learning approach reduces overestimation bias, enhances the stability and accuracy of value function estimates, and improves overall learning [47,48].

Next, we need to adjust the temperature parameter α. A larger value of α increases stochasticity for exploration, while a gradually decreasing temperature encourages more deterministic actions as the policy converges, focusing on exploiting the learned optimal actions. Thus, we adjust α by minimizing the following function:

(31) $$j\left(\alpha \right)=E_{A_{t}\sim \pi }\left[-\alpha \log\pi \left(A_{t}\right|S_{t})-\alpha \bar{H}\right],\;\;\;$$

where $\bar{H}$ represents the entropy threshold.

Finally, the target network weights were updated by considering the moving average of the critic network weights. Specifically, the target network parameters $\theta_{Q_{i}^{\prime }}$ are updated as

(32) $$\theta_{Q_{i}^{\prime }}\leftarrow \varpi \theta_{Q_{i}}+\left(1-\varpi \right)\theta_{Q_{i}^{\prime }}\;,$$

where ϖ  denotes a parameter that controls the update rate. This method of updating the target network weights helps stabilize the training process by smoothing out changes in the network parameters.

### 5.2. Caching Management Subproblem

Efficient caching management is essential for reducing task redundancy and improving resource utilization in UAV-aided MEC. This study adopts a hybrid LFU-LRU caching strategy to dynamically store and manage task-results. The proposed method ensures that frequently requested tasks remain in the cache, while preserving recently accessed tasks, thereby making the system adaptable to different request patterns.

*Caching management strategy*: When the cache capacity constraint is reached, the MEC server cannot store all computed results. Therefore, a cache replacement decision is required. In the proposed framework, the cached result to be erased is determined by the hybrid LFU-LRU cache score. Each cached task-result entry is assigned a hybrid cache score based on two factors: (1) Access frequency $\left(F(T_{n})\right)$, which represents the cumulative number of cache hits recorded for the cached task-result entry since it was inserted into the cache. Specifically, each cached entry maintains an access counter, which is incremented whenever an incoming request matches the cached deterministic task-result (i.e., the same task with the same input) and the cached result is successfully reused. Consequently, computation results with higher access frequencies are regarded as more popular. This corresponds to the LFU component. (2) Last access time $\left(L(T_{n})\right)$, which records the most recent cache hit time of the cached task-result entry and therefore reflects the recency of usage. This corresponds to the LRU component. The cache score is computed as follows:

(33) $$H\left(T_{n}\right)=\delta F\left(T_{n}\right)+\left(1-\delta \right)\frac{1}{t-L\left(T_{n}\right)},\;\;\;\;$$

where δ∈[0, 1] denotes the weighting parameter balancing LFU and LRU. A higher δ favors frequent tasks (LFU). A lower δ prioritizes recently used tasks (LRU). Moreover, $F\left(T_{n}\right)$ is updated upon every cache hit, whereas $L\left(T_{n}\right)$ is updated to the current time whenever the cached task-result is accessed. When the cache reaches its storage capacity, the cached task-result entry with the lowest hybrid score is evicted first because it has the lowest combined priority in terms of access frequency and recency. The newly computed task-result is then inserted into the cache if it was executed at a UAV or the BS.

The weighting factor δ is adapted dynamically based on the observed task repetition rate within a sliding time window of size W. The adaptation rule is defined as:

(34) $$\delta \left(t+1\right)=clip\left(\delta \left(t\right)+\eta \left(r_{t}-r_{th}\right),\;\delta_{min},\delta_{max}\right),\;\;\;\;$$

where η>0 is the adaptation step size, $r_{t}$ denotes the task repetition rate at time step *t*, $r_{th}$ is a predefined repetition rate threshold, and $\delta_{min}$ and $\delta_{max}$ ∈ [0, 1] are the lower and upper bounds of δ, respectively. When $r_{t}>r_{th}$, indicating that task requests are highly repetitive, δ increases, placing more weight on access frequency (LFU) to retain popular tasks in the cache. Conversely, when $r_{t}<r_{th}$, indicating diverse and non-repetitive requests, δ decreases, placing more weight on recency (LRU) to retain the most recently used tasks. This adaptive mechanism ensures that the caching policy self-tunes to the observed request distribution without requiring manual tuning or prior knowledge of the workload.

### 5.3. Joint Offloading and Resource Allocation with Caching (JORC) Scheme

The JORC scheme optimizes computational task execution in UAV-aided MEC networks. It uses an SAC-driven DRL method for offloading and resource allocation while managing caching with a hybrid LFU-LRU mechanism, as depicted in Algorithm 2.

*Working process of JORC algorithm*: The algorithm operates in three main steps:

i. Task handling and cache checking: When a user requests a task, the system first checks whether the result already exists in the cache. If a task-result is found in the cache (cache hit), it is directly returned to the user. If the result is unavailable in the cache (a cache miss), the system proceeds with offloading and resource allocation.

ii. SAC-based offloading and resource allocation: The SAC algorithm is used to determine whether a task should be processed locally, at a UAV or BS. SAC dynamically allocates computing and communication resources to balance the network load and minimize delays. Once the task is processed, the result is delivered to the requesting user.
**Algorithm 2.** JORC algorithm integrating SAC-based offloading and resource allocation with hybrid LFU-LRU caching mechanism.**Input:** Set of tasks $T_{n}$, network state (channel conditions, congestion, and available resources), cache state (stored tasks, access frequencies, last access times, and capacity), trained SAC model for offloading and resource allocation, and initial repetition rate threshold $r_{th}$ for cache adaptation.
**Output:** Updated cache storage, updated frequency and last access time for cached tasks, adapted δ for dynamic cache management. *# Initialization* 1: Define cache storage $S_{m}$ with a maximum capacity $S_{m}^{max}$. 2: Initialize frequency counter $F\left(T_{n}\right)=0$ for all tasks. 3: Initialize the last access time $L\left(T_{n}\right)=0$ for all tasks. 4: Initialize the repetition rate threshold $r_{th}$. *# Task request handling* 5: **For** each requested task $T_{n}$: 6:    Check the result of $T_{n}$ in $S_{m}$ 7:    **If** Cache Hit (Task Found in Cache): 8:     Serve the task-result from cache storage $S_{m}$. 9:     Update access frequency: $F\left(T_{n}\right)\leftarrow F\left(T_{n}\right)+1$. 10:     Update last access time: $L\left(T_{n}\right)\leftarrow t$. 11:   **If** Cache Miss (Task Not in Cache):      #*Need to take offloading decision and resource allocation to process the task* 12:     Execute Algorithm 1 13:     Processed the task as per the decision and obtained the result. 14:     **If** executed at UAV or BS: 15:       **If** cache storage is full: 16:       Compute hybrid cache scores for all cached tasks using Equation (33). 17:       Identify the task with the lowest score:           $\;T_{evict}=arg\underset{T_{j}\in S_{m}}{\min}H\left(T_{n}\right)$ 18:       Remove $T_{evict}$ from cache. 19:     Store new task $T_{n}$ in cache. 20:     Initialize $F\left(T_{n}\right)=1$ and $L\left(T_{n}\right)=t$.  *# Dynamic cache adaptation* 21: Update δ by Equation (34): $\delta \left(t+1\right)=clip\left(\delta \left(t\right)+\eta \left(r_{t}-r_{th}\right),\;\delta_{min},\delta_{max}\right)$


iii. Hybrid LFU-LRU caching mechanism: If the caching space is available, the newly processed task-result is stored. If the cache is full, the algorithm removes the least important task by using a hybrid LFU-LRU strategy. The LFU retains frequently requested tasks, and the LRU ensures that recently used tasks are retained. A weighting factor (δ) adaptively balances the importance of frequency and recency based on user request patterns. This adaptive caching mechanism ensures efficient storage use while reducing redundant computations.

### 5.4. Complexity Analysis

The computational complexity of the proposed JORC framework is analyzed by evaluating the complexity of its key components: the SAC-based offloading and resource allocation algorithm (Algorithm 1) and the caching management process (Algorithm 2).

The SAC algorithm operates over $n_{e}$ episodes, each consisting of $n_{s}$ time steps. The complexity of the algorithm is analyzed as follows: Network initialization and parameter setup: O(1), selecting and executing actions: O(1), storing transitions in the replay buffer: O(1), sampling a mini-batch of size *k* from the replay buffer: O(k), and updating critic and actor parameters using gradient descent: O(1). For each time step, the complexity is O(k). Because each episode consists of $n_{s}$ steps, the complexity for one episode is $O(n_{s}\cdot k)$. Over $n_{e}$ episodes, the total complexity is $O(n_{e}\cdot n_{s}\cdot k)$. Because *k* (mini-batch size) is a small constant, the overall complexity simplifies to: $O(n_{e}\cdot n_{s})$. This complexity reflects the training process of the SAC algorithm, where each iteration involves policy evaluation, policy improvement, and entropy tuning, supporting adaptive offloading and resource-management decisions.

The caching mechanism integrates hybrid LFU and LRU strategies. The complexity of its key operations is: Cache hit update: O(1), computing the hybrid cache score: O(1), eviction process (removing the lowest-score task): O(logC) (if a priority queue is used, where *C* is the cache size), and insertion of a new task into the cache: O(1). Because cache operations involve maintaining a priority queue for eviction, the overall complexity of caching management is O(logC) per caching update. This ensures efficient cache operations with minimal computational overhead.

Therefore, the overall complexity of the JORC scheme combines the SAC-based offloading and resource allocation algorithm with the caching mechanism. Because the caching operations are performed at each time step, the total complexity is: $O(n_{e}\cdot n_{s}\cdot \log C)$.

The primary computational overhead of the proposed framework occurs during the offline training stage of the SAC algorithm. Once training is completed, online decision making only requires a forward pass through the trained actor network, enabling efficient real-time task offloading and resource allocation decisions. As the number of users and the number of UAVs increase, the state and action dimensions also increase, resulting in higher training cost and memory requirements. Nevertheless, the online computational cost remains substantially lower than repeatedly solving the original mixed-integer nonlinear optimization problem for each system state using conventional optimization methods.

## 6. Performance Evaluation

This section assesses the performance of the proposed JORC framework. Simulation experiments were performed to examine its effectiveness in a UAV-aided MEC environment. First, we present the simulation setup, and outline the network environment and parameter configurations. Next, we introduce the comparison baselines and explain why they are relevant for evaluating the JORC. Subsequently, we define the performance metrics used in the evaluation. Finally, we present and analyze the results, demonstrating how JORC enhances the system performance compared to existing approaches.

### 6.1. Simulation Setup

*System setup:* The simulation scenario consisted of one BS positioned at (0, 0) and two rotary-wing UAVs operating at an altitude of 100 m. The users were randomly distributed across a 2 km × 2 km coverage area, as shown in Figure 1. Each user is served by one UAV within its communication range, whereas all UAVs stay inside the BS coverage region. The user positions are refreshed at every time step to reflect their mobility. In each timeslot, a single user generates a single computational task. The task parameters were randomly assigned within the following ranges: task size $D_{n}^{in}$ was randomly set between 2 and 10 Mbit, task complexity $W_{n}$ was set between 600 and 750 cycles per bit, and the maximum tolerable latency $L_{n}^{max}$ was set between 1 and 7 s. The computation capacity of user devices is heterogeneous, randomly set between 0.5 GHz and 0.75 GHz. The UAVs are considered homogeneous, each with a computation capacity of 10 GHz, whereas the BS has a higher computation capacity of 50 GHz for handling more intensive workloads. Additional simulation parameters are listed in Table 3 [9,14,22,35,45,47,48].

*Simulation platform:* The proposed SAC-based JORC framework was developed in Python 3.7 using PyTorch 1.7. The neural networks for both policy and Q functions were designed as fully connected networks with three hidden layers, each containing 400 neurons per layer. Training was performed using the Adam optimizer with a learning rate of $1\times 10^{-4}$, and the ReLU activation function was applied to all the layers. The discount factor was fixed at γ = 0.8, and the model was updated every 100 time slots to ensure stability and efficient learning. The target-update parameter was set to 0.005, the replay buffer size was $10^{6}$ transitions, and the batch size was set to 64. The temperature parameter α, which controls the exploration–exploitation trade-off through entropy regularization, was initialized to $\alpha_{0}=0.2$ and adjusted automatically during training according to (31). The target entropy $\bar{H}\;$ was set to the standard convention $\bar{H}=-5N$, the dimensionality of the joint action space, comprising three continuous offloading-preference scores per user, corresponding to local, assigned-UAV, and BS execution, plus one power allocation value and one CPU frequency allocation value per user, summed over the N active user devices in the scenario. The temperature parameter was updated using the same Adam optimizer and learning rate (1 × 10^−4^) as the actor and critic networks. For each environment step, a single gradient update was performed [9,22,45,47,48].

The complete training procedure is summarized in Algorithm 1. During each episode, the agent interacts with the simulated environment for $n_{s}$ time steps, stores the resulting transitions in the replay buffer, and updates the critic networks, actor network, and temperature parameter after each environment step using randomly sampled mini-batches. The target critic networks are then softly updated according to (32).

The hyperparameter settings for SAC were primarily adopted from the original SAC algorithm and widely used implementations [45,47,48]. Likewise, the hyperparameters of PPO, DDPG, and A3C followed their respective original publications and widely adopted implementations [9,21,22,29,48]. No extensive algorithm-specific hyperparameter tuning was performed. Instead, all algorithms were evaluated under the same problem formulation, simulation environment, training protocol, and evaluation methodology. To ensure a fair comparison, all methods employed the same backbone architecture comprising three hidden layers with 400 neurons each, while retaining their standard algorithm-specific output layers and network components. Unless otherwise required by the original algorithm, all networks were trained using the Adam optimizer with the same learning rate and evaluated using the same ten independent random seeds protocol.

To improve the statistical reliability of the evaluation, all experiments were repeated using ten independent random seeds. The reported results represent the mean performance over the ten runs, and the error bars in Figures 4–15 denote the corresponding 95% confidence intervals. In addition, paired Wilcoxon signed-rank tests were performed between JORC and each baseline across the ten random seeds for all reported metrics. The results confirm that the performance improvements of JORC are statistically significant (*p* < 0.05).

### 6.2. Comparison Baselines

To assess the effectiveness of the proposed JORC framework, we compared it to four baseline schemes. These baselines included different DRL approaches for task offloading and resource allocation in UAV-BS collaborative networks. The selected baselines were as follows:

*(a) DDPG with Caching:* Deep deterministic policy gradient (DDPG) follows an actor–critic DRL structure and is suitable for continuous action-control problems. It is widely used for optimization problems in MEC networks. This baseline applies DDPG with caching, allowing a comparison with JORC in terms of the decision making efficiency and caching effectiveness.

*(b) A3C with Caching:* Asynchronous advantage actor–critic (A3C) is another actor–critic DRL method that improves training stability by using multiple agents in parallel. This baseline evaluates whether asynchronous learning provides advantages over JORC’s SAC-based approach.

*(c) PPO with Caching:* Proximal policy optimization (PPO) is a policy-gradient DRL method that improves decision making using clipped objective functions to prevent drastic updates. This baseline assesses how a policy-based approach with caching compares to JORC’s entropy-regularized actor–critic approach.

*(d) SAC without Caching:* This baseline implements SAC without caching, indicating that task offloading and resource allocation are optimized, but no caching mechanism is applied. To ensure a fair and controlled comparison, SAC without caching baseline uses an identical neural network architecture (three hidden layers with 400 neurons each), the same hyperparameters (learning rate, discount factor, replay buffer size, and batch size), and the same training procedure as JORC. The only difference is that the caching module is entirely disabled (i.e., no cache lookup is performed before task execution), and no task-results are stored after execution. This controlled configuration separates the effect of the caching module from the remaining components of JORC.

Additionally, we evaluated the performance of JORC’s hybrid LFU-LRU caching mechanism by comparing it with three standard cache replacement strategies:

*(a) LFU (Least Frequently Used):* stores tasks that are requested most often.

*(b) LRU (Least Recently Used):* retains the most recently accessed tasks.

*(c) Random:* replaces cached items randomly without considering frequency or recency.

These cache replacement baselines were used to demonstrate the adaptability and effectiveness of JORC’s hybrid LFU-LRU caching policy under different request patterns.

The baseline methods were selected according to the motivation and problem formulation of this work. The primary objective of JORC is to jointly optimize task offloading, computational resource allocation, and task-result caching within a collaborative UAV-BS MEC architecture. Accordingly, our performance evaluation was designed not only to assess the overall effectiveness of the proposed framework but also to isolate the contribution of its major design components under a common problem formulation and system configuration.

Specifically, PPO, DDPG, and A3C were selected as representative actor–critic DRL algorithms because they are widely adopted for continuous optimization in MEC-related studies [28,29,32]. In contrast, value-based methods such as DQN and DDQN require substantial action discretization to handle the continuous power and CPU frequency allocation decisions considered in this work, making them less suitable for the proposed problem formulation. SAC without caching was included to quantify the contribution of the proposed task-result caching mechanism, while conventional LFU, LRU, and random replacement strategies were adopted to evaluate the proposed hybrid LFU-LRU cache replacement policy.

Although several recent UAV-assisted MEC studies investigate related optimization problems, they generally focus on different objectives, such as UAV trajectory optimization, service or content caching, user association, or federated-learning-based caching, rather than task-result caching. Consequently, directly comparing with these methods would simultaneously introduce differences in optimization objectives, caching semantics, and system architectures, making it difficult to isolate the contribution of the proposed framework. Therefore, these representative studies are discussed qualitatively in Section 2 and Table 1, while the quantitative evaluation employs controlled baselines under a common problem formulation and system configuration.

### 6.3. Performance Metrics

To assess the JORC framework, the following performance metrics were considered.

*(a) Average task completion latency:* This metric measures the average time required to complete a task, including transmission, processing, and caching delays. A lower average task completion latency indicates faster task execution, which is crucial for latency-sensitive services.

*(b) Average energy consumption:* This metric assesses the average energy used by a system to complete a single task, including task transmission, computations at UAVs and BS, and caching operations. Minimizing the mean energy consumption helps improve the operational efficiency of UAV-aided MEC networks, because UAVs rely on limited power resources.

*(c) Average cost:* This metric represents the average cost of processing a task, calculated as the weighted combination of latency and energy consumption. Minimizing the average cost is important for improving the overall system performance, because it balances the latency–energy tradeoff.

*(d) Successful task completion ratio (STCR):* This metric measures the proportion of tasks completed within a given time and resource constraints. A higher successful task completion ratio reflects the system’s capability to process tasks reliably, which is necessary for latency-sensitive applications.

*(e) Offloading ratio:* This metric indicates the proportion of tasks offloaded from the local device to external resources, such as UAVs or edge servers for processing. A higher offloading ratio suggests efficient utilization of network resources and helps reduce the local device load, thereby improving overall system efficiency.

*(f) Cache Hit Ratio:* This metric represents the percentage of tasks that are retrieved from the cache without requiring recomputation. A higher cache hit ratio reduces the communication overhead and computational load, thereby improving the overall system efficiency.

These performance metrics provide a comprehensive evaluation of JORC by assessing the processing speed, energy efficiency, caching effectiveness, and overall system performance.

### 6.4. Results and Observations

In this subsection, we discuss our extensive simulation results and findings in detail.

#### 6.4.1. Convergence

**Figure 3:**
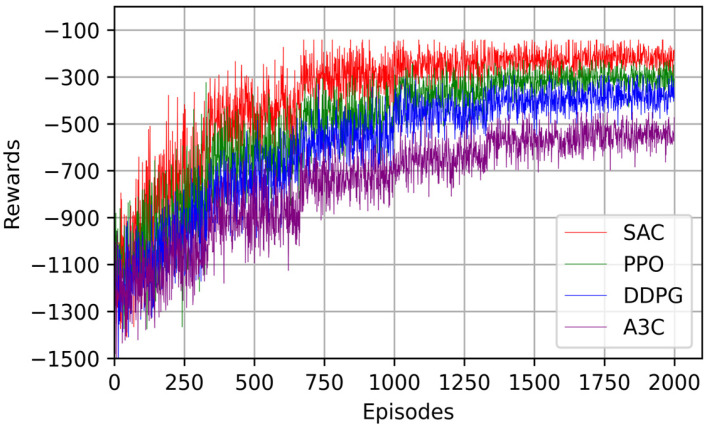
Convergence of SAC and three other DRL-based baseline algorithms.

To examine the convergence pattern of the proposed JORC algorithm, we analyzed SAC’s learning curve against those of the three baseline DRL algorithms: PPO, DDPG, and A3C. The algorithms were trained for 2000 episodes, and Figure 3 presents the change in their performance across training episodes. As shown in Figure 3, SAC converges faster than the other algorithms. It stabilizes at approximately 670 episodes and achieves higher average rewards, reaching approximately −230. This rapid convergence and superior performance highlight SAC’s effectiveness for the given task. In contrast, PPO and DDPG converged more slowly. Both algorithms stabilize at approximately 1000 episodes, with PPO reaching an average reward of approximately −320 and DDPG at approximately −380. These algorithms take longer to achieve stable performance, and their rewards are lower than those of SAC. A3C shows the slowest convergence of all the algorithms. It takes approximately 1350 episodes to stabilize, with an average reward of approximately −530. Although A3C eventually stabilized, its performance was significantly lower than those of SAC, PPO, and DDPG. Overall, SAC outperformed the other algorithms, demonstrating both faster convergence and higher average rewards, making it a stronger choice for the tasks at hand.

#### 6.4.2. Performance Metrics Evaluation

The performance of the proposed JORC framework is evaluated against the baseline DRL methods, PPO, DDPG, A3C (with caching), and SAC (without caching), using four performance metrics: task completion latency, energy consumption, cost, and the STCR. To evaluate the robustness and scalability of the proposed framework, experiments were conducted under different system configurations by varying the number of user devices, the number of UAVs, the UAV cache capacity, and the BS cache capacity, while keeping the remaining system parameters fixed.

**Figure 4:**
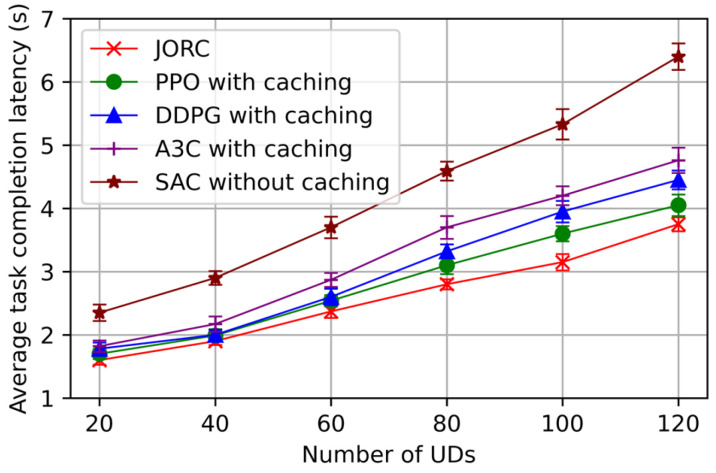
Average task completion latency vs. the number of UDs.

**Figure 5:**
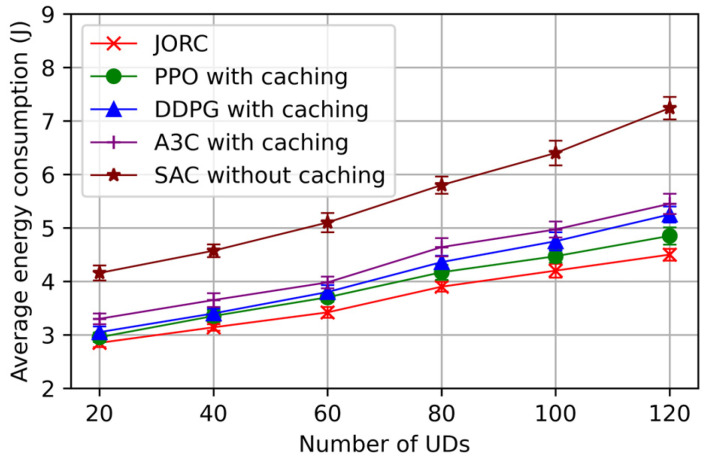
Average energy consumption vs. the number of UDs.

**Figure 6:**
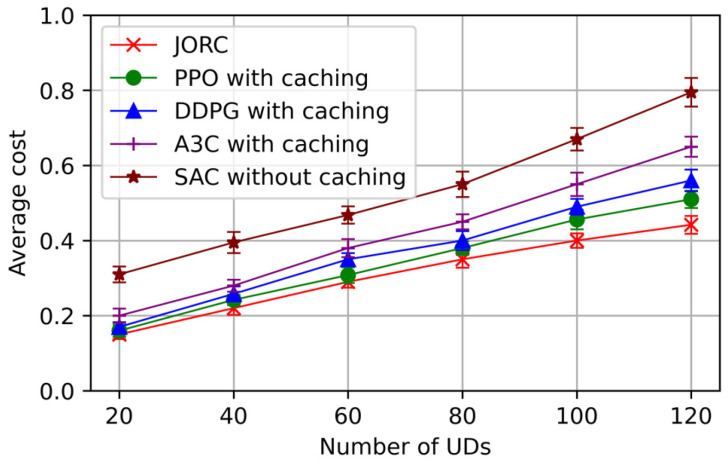
Average cost vs. the number of UDs.

**Figure 7:**
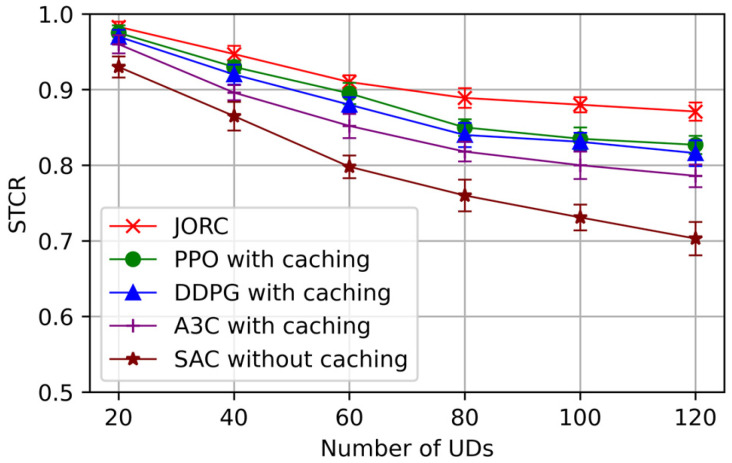
Successful task completion ratio vs. the number of UDs.

Figures 4–7 and 12 present the performance of the four metrics as functions of the number of user devices, with the number of UAVs fixed at two.

Figure 4 shows the average task-completion latency as a function of the number of UDs. As expected, the latency increased with the number of UDs for all methods. However, JORC consistently outperformed the other methods. At 20 UDs, JORC achieves a latency of approximately 1.6 s, whereas at 120 UDs, the latency increases to 3.7 s. In comparison, SAC without caching showed a latency of approximately 2.4 s at 20 UDs, which increased to approximately 6.4 s at 120 UDs, the highest among all methods. PPO, DDPG, and A3C with caching show similar performance, with latencies ranging from 1.7 to 2 s at 20 UDs and 4 to 5 s at 120 UDs. These results confirm that the JORC maintains the lowest latency across all UDs, demonstrating its superior performance in managing task completion times even as the number of UDs increases.

Figure 5 illustrates the energy consumption as a function of the number of UDs. At 20 UDs, JORC consumes approximately 2.8 Joules, whereas SAC without caching requires approximately 4.1 Joules. As the number of UDs increased to 120, JORC’s energy consumption rose to 4.55 Joules, but it remained the most energy efficient. However, SAC without caching increased to approximately 7.2 Joules, showing the highest energy consumption. PPO, DDPG, and A3C with caching methods exhibited moderate energy consumption, with values between 3 and 3.55 Joules at 20 UDs, and 4.8 to 5.5 Joules at 120 UDs. These results highlight that JORC is the most energy-efficient solution, effectively managing energy consumption even as the number of UDs increases.

The average cost per task is shown in Figure 6. At 20 UDs, JORC incurs a cost of approximately 0.15, which increases to 0.43 at 120 UDs. By contrast, SAC without caching starts at 0.31 at 20 UDs and increases to 0.8 at 120 UDs, making it the most cost-inefficient method. PPO, DDPG, and A3C with caching exhibit costs ranging from 0.16 to 0.2 at 20 UDs and 0.5 to 0.7 at 120 UDs. This analysis indicates that JORC remains the most cost-efficient approach across different numbers of UDs, effectively minimizing the cost of task processing even at the system scale.

Figure 7 depicts STCR as a function of the number of UDs. At 20 UDs, JORC achieved an STCR of 0.98, where SAC without caching reached only 0.92. As the number of UDs increased, the STCR decreased for all methods. At 120 UDs, JORC maintained a high STCR of 0.88, whereas SAC without caching dropped to 0.7, which was the lowest among all methods. PPO, DDPG, and A3C with caching show moderate performance, with STCRs ranging from 0.78 to 0.85 at 120 UDs.

**Figure 8:**
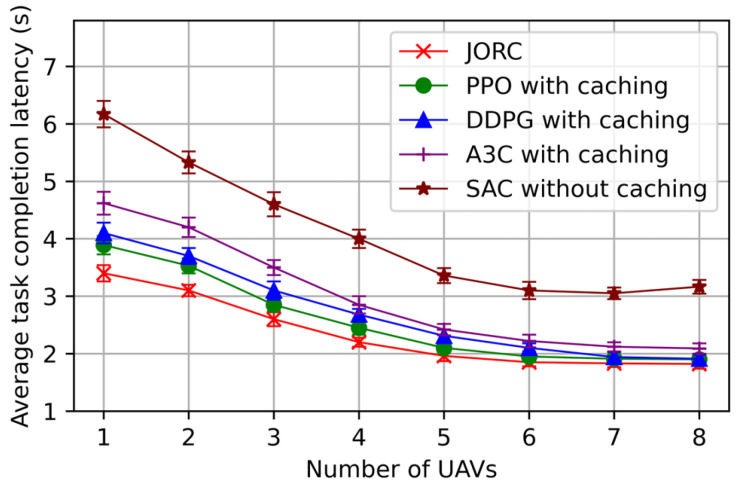
Average task completion latency vs. the number of UAVs.

**Figure 9:**
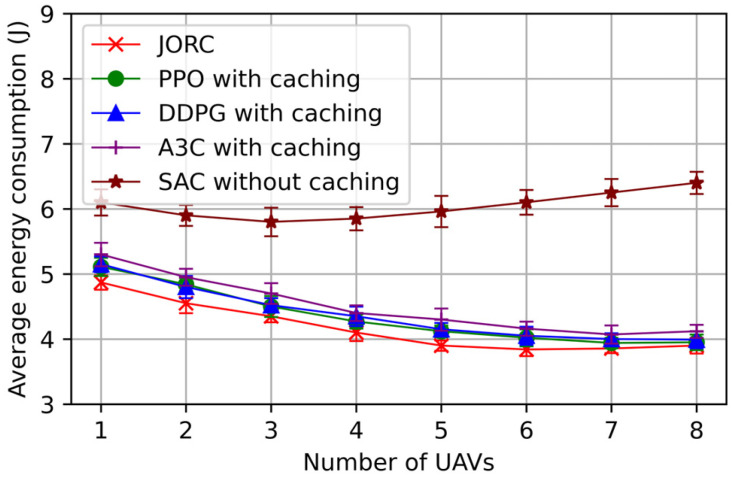
Average energy consumption vs. the number of UAVs.

**Figure 10:**
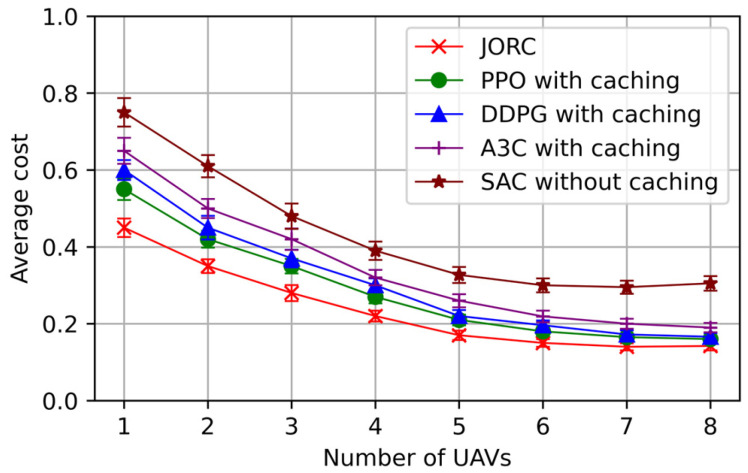
Average cost vs. the number of UAVs.

**Figure 11:**
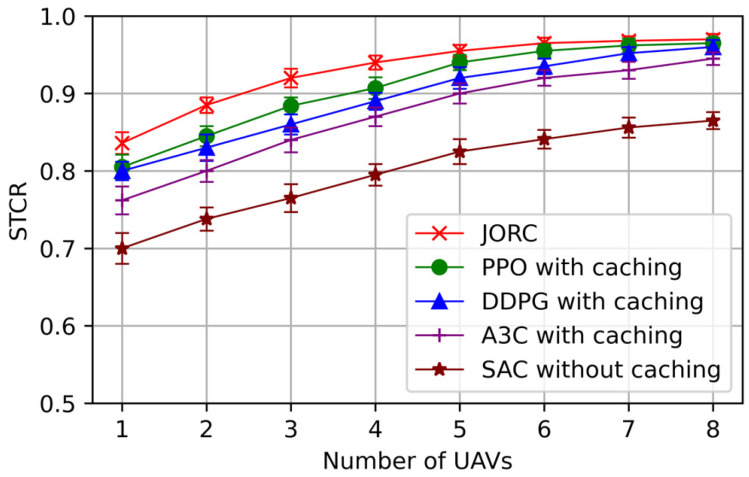
Successful task completion ratio vs. the number of UAVs.

Next, the robustness and scalability of the proposed framework are evaluated by varying the number of UAVs while fixing the number of user devices at 100, as shown in Figures 8–11 and 13.

Figure 8 illustrates the variation in average task completion latency as the number of UAVs increases from one to eight. As expected, latency decreases with the addition of UAVs, benefiting from more efficient task distribution and the expanded caching capacity. Specifically, with a single UAV, JORC achieves a latency of approximately 3.4 s, outperforming PPO, DDPG, A3C (with caching), and SAC (without caching). As the number of UAVs increases to five, JORC’s latency significantly reduces to around 1.8 s, maintaining the lowest latency among all methods. However, beyond five UAVs, the improvements in latency become marginal, with JORC stabilizing at approximately 1.7 s from 6 to 8 UAVs. A similar trend of diminishing returns is observed for the other methods, although their latencies remain consistently higher than JORC’s. In contrast, SAC without caching consistently shows higher latency, with a slight increase in latency beyond seven UAVs, likely due to suboptimal task distribution in the absence of caching mechanisms. These findings suggest that, while increasing the number of UAVs enhances system performance, the benefits saturate beyond a certain point, suggesting an optimal range for UAV deployment.

Figure 9 depicts the relationship between the number of UAVs and average energy consumption. As the number of UAVs increases, energy consumption generally decreases due to the enhanced task distribution and greater cache availability, reducing unnecessary transmissions, and computational load. JORC consistently exhibits the lowest energy consumption across all methods. For instance, with a single UAV, JORC consumes about 4.9 J, which decreases to approximately 3.9 J when five UAVs are deployed. Beyond this point, the improvement in energy efficiency becomes negligible, with consumption stabilizing, indicating a saturation threshold. A similar leveling off is seen in PPO, DDPG, and A3C with caching, though these methods consistently consume more energy than JORC at every UAV count. In contrast, SAC without caching shows the highest energy consumption, with a gradual increase after four UAVs, likely due to inefficient task offloading in the absence of caching. These results highlight that, while increasing the number of UAVs initially enhances energy efficiency, the benefit stagnates, suggesting that five UAVs is an optimal number for minimizing energy consumption in the given condition.

Figure 10 illustrates the average task completion cost as the number of UAVs increases from one to eight. The cost, representing a weighted combination of latency and energy consumption, decreases steadily for all methods as more UAVs are deployed. JORC consistently achieves the lowest average cost, reducing from approximately 0.46 with a single UAV to around 0.17 with six UAVs. Beyond this point, the cost stabilizes, showing diminishing returns. PPO, DDPG, and A3C with caching also exhibit a downward trend, but their costs remain slightly higher than JORC’s, stabilizing between 0.18 and 0.2 at eight UAVs. In contrast, SAC without caching starts with the highest cost, around 0.75 at a single UAV, and shows minimal improvement after six UAVs. These findings suggest that, while adding UAVs initially improves cost efficiency, the benefit levels off after six UAVs, indicating that six UAVs is an optimal point for efficient deployment in the given condition.

Figure 11 depicts the STCR as a function of the number of UAVs. As the number of UAVs increases, the STCR improves for all methods, reflecting more efficient task management and a reduced system overload. JORC consistently achieves the highest STCR across all UAV counts, starting at approximately 0.84 with a single UAV and steadily rising to around 0.965 with five UAVs. Beyond five UAVs, the improvement becomes minimal, with STCR stabilizing near 0.97 at eight UAVs. PPO, DDPG, and A3C with caching also show steady improvement, reaching STCR values between 0.93 and 0.96 at eight UAVs, but remain slightly lower than JORC. In contrast, SAC without caching starts with the lowest STCR of about 0.70 and only reaches 0.86 at eight UAVs, indicating inefficiency without caching. These results suggest that, while increasing the number of UAVs improves the task success rate, the gains level off beyond five UAVs, highlighting an optimal point for maximizing STCR in the given condition.

**Figure 12:**
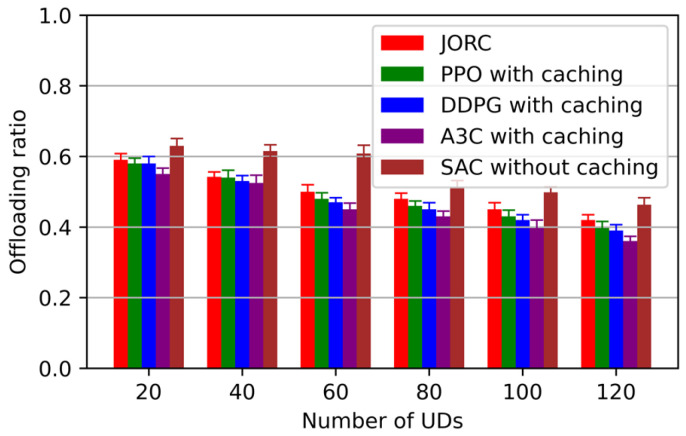
Offloading ratio vs. the number of UDs.

**Figure 13:**
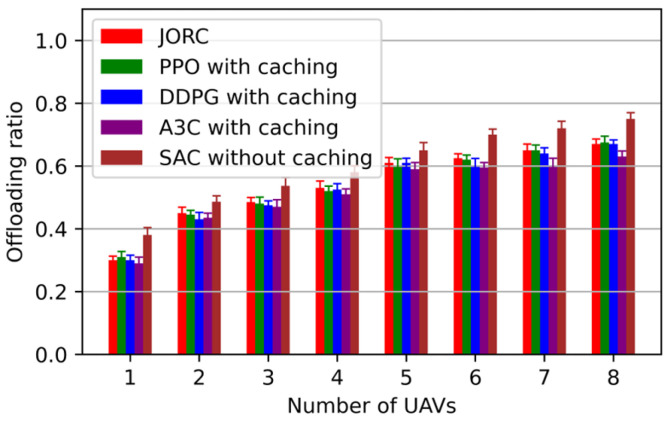
Offloading ratio vs. the number of UAVs.

Figure 12 and Figure 13 illustrate the offloading ratio vs. number of UDs and UAVs. As shown in Figure 12, as the number of UDs increases from 20 to 120 (where the number of UAVs is fixed at 2), the offloading ratio tends to remain relatively stable for all methods, with minor fluctuations. JORC achieved the highest offloading ratio across all UDs. The offloading ratio for JORC is nearly identical across the whole range, indicating its robustness in task offloading regardless of the load from the UDs. PPO, DDPG, and A3C, all using caching mechanisms, follow closely behind JORC, with offloading ratios ranging from 0.4 to 0.6 across the UDs. Although these methods show a slight decrease as the number of UDs increases, their performance remains strong in comparison with that of SAC. SAC without caching exhibited the highest offloading ratio at all UDs, peaking at approximately 0.6 for smaller numbers of UDs and gradually dropping below 0.6 as the load increased. The significant gap between SAC without caching and the other methods highlights the importance of caching in improving the task offloading efficiency.

Figure 13 depicts the offloading ratio as a function of the number of UAVs, ranging from one to eight (where the number of UDs is fixed at 100). As expected, the offloading ratio increases with the number of UAVs due to both the enhanced computing capacity and the improved task distribution. JORC, PPO, DDPG, and A3C (all with caching) show similar trends, with the offloading ratio steadily improving as the number of UAVs increases. However, SAC without caching consistently shows the highest offloading ratio across all UAV counts. This is because SAC without caching lacks a caching policy, resulting in no cache hits and, thus, every task must either be computed locally or offloaded to the UAV or BS. In contrast, methods with caching (such as JORC, PPO, DDPG, and A3C) show lower offloading ratio compared to SAC without caching, highlighting the benefit of the caching mechanism in reducing communication and computation overhead. These results emphasize that incorporating caching strategies results in more efficient task management by decreasing the need for redundant computations and offloading, thus reducing the overall system load.

**Figure 14:**
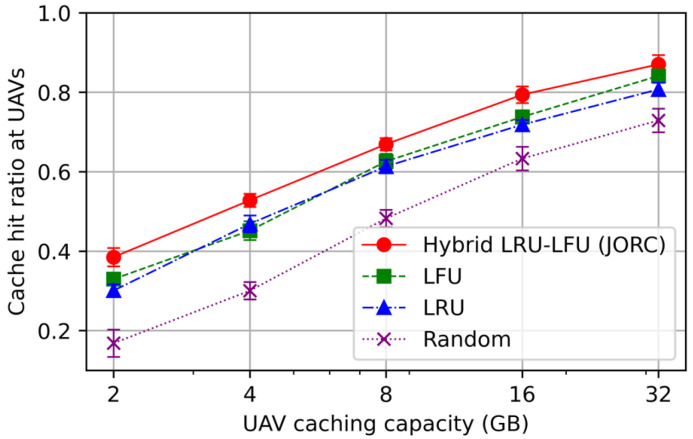
Cache hit ratio vs. UAV caching capacity.

**Figure 15:**
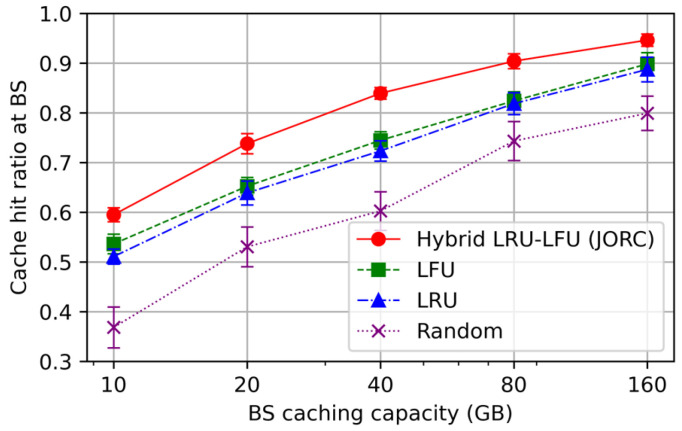
Cache hit ratio vs. BS caching capacity.

Finally, the impact of caching resources is evaluated by varying the UAV and BS cache capacities. Figure 14 and Figure 15 present the cache hit ratio versus the UAV and BS cache capacities, respectively, for a network with two UAVs, one BS, and 100 UDs.

Figure 14 shows the cache hit ratio of the UAVs as a function of caching capacity. The figure illustrates that the cache hit ratio improves as the caching capacity increases for all methods. JORC consistently achieves the highest cache hit ratio, starting at 0.4 for a 2 GB caching capacity and reaching 0.9 at a 32 GB caching capacity. This clear upward trend demonstrates the effectiveness of the Hybrid LRU-LFU (JORC) caching strategy in improving the cache performance as the available storage capacity increases. The LFU and LRU caching mechanisms showed a similar upward trend, but with lower cache hit ratios. LFU increases from 0.33 to approximately 0.82 as caching capacity grows, while LRU progresses from 0.3 to approximately 0.8 at 32 GB. Random caching exhibited the lowest cache hit ratio, starting at 0.18 and only reaching 0.72 at the highest caching capacity. These results emphasize the superior performance of JORC, which significantly outperformed the other methods, particularly as the caching capacity increased.

Figure 15 shows the cache hit ratios at the BS for various caching capacities. Similarly to the UAV results, the cache hit ratio improved with an increase in caching capacity. JORC once again leads with the highest hit ratio, which starts at 0.6 for 10 GB and reaches approximately 0.95 at 160 GB. LFU and LRU show similar but lower trends, with LFU ranging from 0.53 to 0.9 and LRU ranging from 0.51 to 0.89 at the highest caching capacity. Random caching showed the least improvement, from 0.38 to 0.8, demonstrating its inefficiency. Figure 14 and Figure 15 show that the Hybrid LRU-LFU (JORC) consistently delivered the highest cache hit ratio for both UAVs and the BS, and the performance of all methods improved with an increase in caching capacity.

## 7. Discussion

This section discusses the key findings of the proposed JORC framework, outlines the assumptions and limitations of the current system model, and identifies several promising directions for future research.

### 7.1. Discussion of Experimental Results

The superior performance of JORC can be attributed to two complementary mechanisms. First, compared with PPO, DDPG, and A3C (all equipped with the same caching mechanism), JORC benefits from SAC’s entropy-regularized off-policy learning, which achieves faster and more stable policy convergence as demonstrated by the convergence results in Section 6.4.1. Consequently, SAC learns more effective task offloading and resource allocation policies than the other DRL algorithms under identical caching support, resulting in consistently lower latency, energy consumption, and overall system cost across the evaluated scenarios. Second, compared with SAC without caching, the additional performance gain originates from the proposed hybrid LFU-LRU task-result caching mechanism. By reusing previously computed task-results whenever cache hits occur, redundant task transmission and computation are substantially reduced, thereby lowering latency, energy consumption, and overall system cost while improving cache utilization.

The performance gains are not uniform across all operating conditions but become more pronounced as the system load increases relative to the available resources. As the number of UDs increases from 20 to 120 (Figure 4, Figure 5, Figure 6 and Figure 7), the performance gap between JORC and SAC without caching gradually widens because a larger user population increases the probability of repeated task requests, enabling the caching mechanism to eliminate an increasing amount of redundant computation. Likewise, the advantage of JORC over the other DRL baselines is most evident when only a small number of UAVs are available (Figure 8, Figure 9, Figure 10 and Figure 11), where communication and computing resources are more constrained and efficient resource management becomes increasingly important. As the number of UAVs increases, additional computing resources reduce resource contention for all methods, causing the performance differences to gradually decrease and eventually approach saturation.

Overall, the experimental results demonstrate that the major components of JORC provide complementary benefits. Comparisons with PPO, DDPG, and A3C under identical caching support validate the effectiveness of SAC for joint task offloading and computational resource allocation, while the comparison with SAC without caching quantifies the additional benefit of the proposed task-result caching mechanism. Furthermore, comparisons with LFU, LRU, and random replacement strategies confirm the effectiveness of the proposed hybrid LFU-LRU cache replacement policy. The robustness of these findings is supported by experiments conducted over ten independent random seeds, with 95% confidence intervals reported in Figure 4, Figure 5, Figure 6, Figure 7, Figure 8, Figure 9, Figure 10, Figure 11, Figure 12, Figure 13, Figure 14 and Figure 15 and paired Wilcoxon signed-rank tests confirming that the performance improvements of JORC are statistically significant (*p* < 0.05).

### 7.2. Model Assumptions and Limitations

To maintain analytical tractability, the proposed framework adopts several simplifying assumptions whose practical implications should be acknowledged.

First, the UAV-assisted MEC architecture models UAVs as lightweight aerial MEC nodes that provide localized and temporary computing support, while the terrestrial BS performs more computationally intensive processing. This collaborative architecture is consistent with the UAV-enabled MEC paradigm reported in the literature, where UAV-mounted MEC servers complement terrestrial infrastructure in scenarios such as communication hotspots, disaster recovery, and areas without reliable terrestrial MEC coverage [49]. The proposed framework explicitly models the communication and computation energy associated with task execution, whereas UAV propulsion energy, battery discharge dynamics, and flight-endurance constraints are beyond the scope of the present study.

Second, the proposed task-result caching mechanism assumes deterministic computation tasks, where the repeated requests with identical task inputs produce identical outputs. This assumption enables efficient task-result reuse and facilitates the joint optimization of task offloading, resource allocation, and task-result caching. However, in practical MEC systems, the cached results may become outdated because of evolving task inputs or changes in the underlying data, requiring cache invalidation and consistency management. Moreover, task identification is assumed to be deterministic, such that cache reuse is triggered only when the same task with identical input is requested. Applications involving continuously varying or similar inputs may therefore require more advanced techniques, such as content-based hashing or similarity-aware matching, which are beyond the scope of the present work.

Third, the communication model considers only large-scale path loss under LoS-dominant and quasi-static assumptions, allowing the analysis to focus on the proposed joint optimization framework. Consequently, small-scale fading and its impact on transmission rates and task offloading decisions are not considered. In addition, the BS and each UAV are assumed to employ single-antenna transmission, consistent with the scalar channel gains in (1) and (2), and therefore do not capture multi-antenna transmission or beamforming techniques. Furthermore, the downlink transmission latency and energy are assumed to be negligible because the output data size is considerably smaller than the input task size and the downlink generally provides a higher transmission rate. Cache lookup is also assumed to be instantaneous and error-free, without considering lookup delay or cache-management overhead. These assumptions are consistent with commonly adopted abstractions in related UAV-assisted MEC studies [14,15,44], and enable the study to focus on the joint optimization problem.

Finally, all the reported results are obtained through simulation and therefore primarily demonstrate the algorithmic behavior of JORC under controlled UAV-assisted MEC conditions. Although simulation enables systematic comparison under identical network settings, it cannot fully capture practical factors such as hardware processing latency, signaling overhead, imperfect state information, cache synchronization, wireless-channel uncertainty, and UAV operational constraints. Furthermore, although our comparisons with the controlled baselines adopted in this work are designed to both isolate the contribution of each major design component and evaluate the overall effectiveness of the integrated JORC framework under a common problem formulation and system configuration, the quantitative evaluation does not include direct comparisons with recent state-of-the-art UAV-assisted MEC optimization frameworks. This represents a limitation of the present experimental evaluation. Consequently, the reported results should be interpreted as evidence of the potential effectiveness of JORC rather than as direct proof of deployment performance.

### 7.3. Future Research Directions

Several promising directions may further extend the proposed JORC framework. On the caching side, future work will investigate more practical task-result caching strategies incorporating cache invalidation, distributed cache-consistency management, evolving task inputs, and similarity-aware or approximate cache matching, enabling semantically similar requests to benefit from cached computation results.

The proposed UAV-assisted MEC framework can also be extended by jointly considering communication, computation, and propulsion energy, together with battery dynamics, flight-endurance constraints, and UAV trajectory optimization, to provide a more realistic representation of UAV operation.

The communication model may be further enhanced by incorporating small-scale fading, shadowing, mobility-induced channel variations, and multi-antenna transmission with beamforming, thereby enabling the joint optimization of beamforming, task offloading, computational resource allocation, and task-result caching under more realistic wireless environments.

Finally, an important direction for future research is the practical validation of the proposed framework through comprehensive runtime benchmarking, scalability analysis under larger network deployments, hardware-in-the-loop experiments, UAV-assisted MEC testbeds, and ultimately real-world deployments. Such studies will provide further insight into the practical applicability, robustness, and deployment performance of JORC under realistic operating conditions.

## 8. Conclusions

This study introduced the JORC framework for improving task execution in UAV-aided MEC networks, specifically for latency-sensitive applications. The framework jointly optimizes task offloading and resource allocation using a SAC-based DRL approach, while incorporating a hybrid LFU-LRU caching mechanism to enhance system efficiency. The proposed scheme effectively improves overall system performance by reducing task execution delays, optimizing resource distribution, and minimizing redundant computations. The simulation results demonstrated that the proposed JORC achieves better performance than existing methods in terms of latency reduction, energy efficiency, cache utilization, and overall cost minimization, making it a practical solution for real-time services, such as AR/VR, autonomous driving, and IoT-based applications. The combination of intelligent offloading and resource allocation and adaptive caching enhances system performance under dynamic network conditions in UAV-aided MEC systems.

## Figures and Tables

**Table 1:** Comparison of the proposed JORC framework with representative related studies.

Ref.	Year	Offloading	Resource Allocation	Caching Type	UAV-BS Collaboration	Solution Method	Contribution/Limitation
[15]	2021	✓	✓	Task result	—	Conventional	No learning-based adaptability; no UAV involvement
[42]	2022	✓	✓	—	—	Conventional	Requires resolving the optimization problem for each network instance
[35]	2022	✓	✓	Task result	—	Meta-RL	Two-tier MEC only; no continuous entropy-regularized control
[36]	2023	✓	✓	Service	—	DDQN	Discrete-action DQN-based control; service caching; no result reuse
[26]	2024	✓	✓	Content	UAV-assisted (IoV)	Federated DRL	No transmit power allocation; reusable task-result caching
[29]	2025	✓	✓	Content	UAVs, no BS	DDPG	Content caching; no BS collaboration
[18]	2026	✓	✓	—	Cooperative multi-UAV	Conventional	No caching component; no collaboration with BS
[40]	2026	✓	✓	Content	Multi-UAVs	DQN	Content caching rather than reusable task-result caching
Proposed JORC	2026	✓	✓	Task result	✓	SAC (entropy-regularized, continuous control) + hybrid LFU-LRU caching	Joint optimization of task offloading, resource allocation, and adaptive task-result caching within a UAV-BS collaborative architecture

Note: “✓” means “considered”, and “—” means “not considered”.

**Table 2:** List of notations used in this study.

Notation	Definition	Notation	Definition
*N*	Set of UDs	$L_{n,m,b}^{off}$	Uplink transmission latency (UAV to BS)
*M*	Set of UAVs	$E_{n,m,b}^{off}$	Uplink transmission energy consumption (UAV to BS)
*H*	UAV altitude	$L_{n}^{UD}$	Latency for computing on UD
$h_{n,m}$	Channel gain	$L_{n}^{UAV}$	Latency for computing at UAV
$d_{n,m}$	Horizontal distance between UD *n* to UAV *m*	$L_{n}^{BS}$	Latency for computing at BS
*B*	Bandwidth	$E_{n}^{UD}$	Energy consumption for computing on UD
$N_{0}$	Noise power	$E_{n}^{UAV}$	Energy consumption for computing at UAV
$P_{n}$	Transmit power of UD *n*	$E_{n}^{BS}$	Energy consumption for computing at BS
$P_{m}$	Transmit power of UAV *m*	$f_{n}$	Computing ability of UD (CPU cycles/s)
$T_{n}$	Task information of UD *n*	$F_{u}$	Computing ability of UAV (CPU cycles/s)
$L_{n}^{max}$	Maximum tolerable latency	$F_{b}$	Computing ability of BS (CPU cycles/s)
$D_{n}^{in},W_{n},$ $\;D_{n}^{out}$	Task size, Computing workload, Result size	k	Energy conversion efficiency
$L_{n,m}^{off}$	Uplink transmission latency (UD to UAV)	$S_{m}^{max}$	Maximum caching capacity of MEC *m*
$E_{n,m}^{off}$	Uplink transmission energy consumption (UD to UAV)	$S_{b}^{max}$	Maximum caching capacity of BS

**Table 3:** Simulation parameters.

Parameter	Value	Parameter	Value
N	100	k	$1\times 10^{-27}$
M	2	$h_{0}$	−50 dB
H	100 m	$S_{u}^{max}$	2 GB
$D_{n}^{in}$	[2, 10] Mbit	$S_{b}^{max}$	10 GB
$D_{n}^{out}$	[0.1, 1] Mbit	$\gamma_{n}^{L}=\gamma_{n}^{E}$	0.5
$W_{n}$	[600, 750] cycles/bit	W	50
$L_{n}^{max}$	[1, 7] s	η	0.05
$E_{n}^{max}$	20 J	$r_{th}$	0.5
B	20 MHz	λ	$1\times 10^{-4}$
$f_{m}$	[0.5, 0.75] GHz	$n_{e}$	$2\times 10^{3}$
$f_{u}$	10 GHz	$n_{s}$	$1\times 10^{3}$
$f_{b}$	50 GHz	ϖ	0.005
$\alpha_{0}$	0.2	$\bar{H}$	−5 N

## Data Availability

The data presented in this study are available upon reasonable request, subject to approval by the funding organization and applicable institutional and copyright requirements. The implementation and simulation framework developed for this study is being considered for public release through a public repository (e.g., GitHub), subject to approval by the funding organization and applicable institutional and copyright requirements.
